# piRNAs initiate transcriptional silencing of spermatogenic genes during *C. elegans* germline development

**DOI:** 10.1016/j.devcel.2021.11.025

**Published:** 2022-01-24

**Authors:** Eric Cornes, Loan Bourdon, Meetali Singh, Florian Mueller, Piergiuseppe Quarato, Erik Wernersson, Magda Bienko, Blaise Li, Germano Cecere

**Affiliations:** 1 Mechanisms of Epigenetic Inheritance, Department of Developmental and Stem Cell Biology, Institut Pasteur, UMR 3738, CNRS, Paris 75015, France; 2 Imaging and Modeling Unit, Institut Pasteur, UMR 3691 CNRS, C3BI USR 3756 IP CNRS, Paris, France; 3 Division of Genome Biology, Department of Medical Biochemistry and Biophysics, Karolinska Institutet, Stockholm 17165, Sweden; 4 Science for Life Laboratory, Tomtebodavägen 23A, Stockholm 17165, Sweden; 5 Bioinformatics and Biostatistics Hub, Department of Computational Biology, Institut Pasteur, USR 3756, CNRS, Paris 75015, France

**Keywords:** piRNAs, small RNAs, RNAi, *C. elegans*, germ granules, transcriptional silencing, epigenetics, spermatogenesis, germline development, fertility

## Abstract

Eukaryotic genomes harbor invading transposable elements that are silenced by PIWI-interacting RNAs (piRNAs) to maintain genome integrity in animal germ cells. However, whether piRNAs also regulate endogenous gene expression programs remains unclear. Here, we show that *C. elegans* piRNAs trigger the transcriptional silencing of hundreds of spermatogenic genes during spermatogenesis, promoting sperm differentiation and function. This silencing signal requires piRNA-dependent small RNA biogenesis and loading into downstream nuclear effectors, which correlates with the dynamic reorganization of two distinct perinuclear biomolecular condensates present in germ cells. In addition, the silencing capacity of piRNAs is temporally counteracted by the Argonaute CSR-1, which targets and licenses spermatogenic gene transcription. The spatial and temporal overlap between these opposing small RNA pathways contributes to setting up the timing of the spermatogenic differentiation program. Thus, our work identifies a prominent role for piRNAs as direct regulators of endogenous transcriptional programs during germline development and gamete differentiation.

## Highlights

• piRNAs trigger transcriptional silencing of spermatogenic genes through HRDE-1
• Nuclear silencing correlates with a reorganization of distinct nuage condensates
• Nuclear silencing is required for sperm differentiation and function
• The Argonaute CSR-1 antagonize piRNA targeting to enable spermatogenic expression

## Graphical abstract

**Figure:**
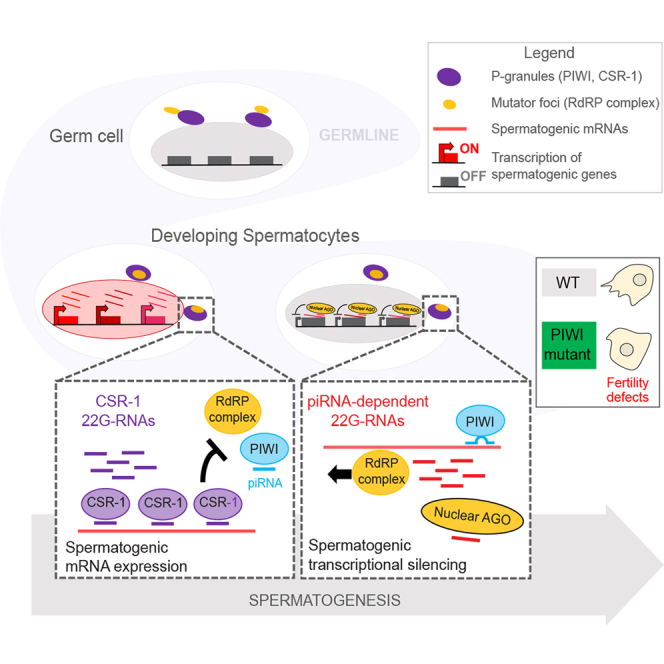

## Teaser

PIWI-interacting RNAs (piRNAs) are known to repress foreign transposable elements in animal germlines. Cornes et al. report that piRNAs trigger the transcriptional silencing of endogenous spermatogenic gene expression program during animal development to promote sperm differentiation and functions.

## Introduction

The RNA-guided targeting of nucleic acids is an ancient and conserved mechanism of cellular immunity that has been evolutionarily adapted and diversified to regulate eukaryotic gene expression. Loaded into Argonaute (AGO) effector proteins, non-coding small RNAs provide targeting specificity for mRNAs through antisense sequence complementarity.

In animal germ cells, PIWI-interacting RNAs (piRNAs) have been extensively characterized as a defense mechanism against transposable elements (TEs) to promote fertility and genome integrity (Ozata et al., 2019). Yet, a large fraction of piRNA sequences in different organisms do not match TEs (Aravin et al., 2006; Shen et al., 2018), and growing evidence points to extended possibilities in gene regulation (Rojas-Ríos and Simonelig, 2018). Furthermore, non-sequence-specific mechanisms of piRNA-mediated gene regulation have also been described (Shen et al., 2018; Vourekas et al., 2016), showing that piRNAs do not necessarily rely on perfect sequence complementarity to function. These features leave piRNAs as a sort of “specificity paradox” in the sequence-based regulation of gene expression, making it difficult to study their direct targets and biological functions.

In the *C. elegans* germline, thousands of highly diverse piRNAs are loaded into the AGO protein PIWI (Batista et al., 2008; Das et al., 2008) to target and initiate the silencing of transcripts from foreign invasive elements such as single-copy transgenes and TEs (Ashe et al., 2012; Bagijn et al., 2012; Shirayama et al., 2012). The mechanism of piRNA-mediated silencing relies on an amplification step that requires RNA-dependent RNA polymerases (RdRPs) and components of the *Mutator* complex to produce secondary antisense small RNAs (called 22G-RNAs) from the targeted transcript (Bagijn et al., 2012; Lee et al., 2012; Luteijn et al., 2012; Shen et al., 2018; Zhang et al., 2018). These piRNA-dependent 22G-RNAs are loaded into downstream nuclear and cytoplasmic worm-specific Argonaute (WAGO) effector proteins, targeting nascent RNAs for transcriptional silencing and mature RNAs for post-transcriptional silencing (Bagijn et al., 2012; Buckley et al., 2012; Lee et al., 2012).

Given that *C. elegans* piRNAs target transcripts by imperfect sequence complementarity (Bagijn et al., 2012; Lee et al., 2012; Shen et al., 2018; Zhang et al., 2018), PIWI/piRNA complexes have been detected promiscuously interacting with most of the germline transcriptome (Shen et al., 2018). This overwhelming targeting capacity contrasts with the limited number of reported examples for direct piRNA silencing on endogenous genes (Shen et al., 2018; Tang et al., 2018). Consequently, whether piRNAs’ functions can be co-opted to regulate endogenous gene expression programs remains an open question. Several mechanisms have been proposed to confer resistance to piRNA-mediated silencing of endogenous germline genes (Frøkjær-Jensen et al., 2016; Seth et al., 2018; Shen et al., 2018; Zhang et al., 2018), including the targeting and licensing of mRNAs by the AGO protein CSR-1. Although CSR-1 targeting can counteract piRNA-mediated silencing of single-copy transgenes (Seth et al., 2013; Wedeles et al., 2013), the relevance of this competition on endogenous germline genes is unclear (Shen et al., 2018; Zhang et al., 2018). Moreover, whether and when the expression of germline genes is vulnerable to or protected from piRNA silencing is unknown.

A well-conserved aspect of germline AGOs and small RNA biogenesis factors is their localization to perinuclear liquid-like condensates present in germ cells, also known as nuage (Voronina et al., 2011). These condensates are enriched in RNAs and RNA-binding proteins and are suspected to regulate post-transcriptional processes necessary for germ cell fate specification and function. The nuage of the *C. elegans* germ cells contains a highly organized and dynamic repertoire of distinct condensates, whose segregated components seem to be functionally linked (Wan et al., 2018). For instance, whereas the Argonautes CSR-1 and PIWI localize to P granule condensates (Chen et al., 2020; Claycomb et al., 2009; Marnik et al., 2019; Wang and Reinke, 2008) at the external face of nuclear pores (Pitt et al., 2000), the 22G-RNA biogenesis machinery required for piRNA-mediated silencing concentrates into spatially distinct condensates, known as *Mutator* foci (Phillips et al., 2012; Uebel et al., 2020; Wan et al., 2018). The spatial separation of PIWI from its downstream machinery required for 22G-RNA biogenesis has been proposed as a mechanism to prevent detrimental piRNA-mediated silencing of endogenous genes (Dodson and Kennedy, 2019; Ouyang et al., 2019). Therefore, how the piRNA-targeting events in P granules trigger the production of 22G-RNAs in *Mutator* foci to achieve transcriptional and post-transcriptional silencing remains unknown.

## Results

### piRNAs directly target spermatogenic genes for transcriptional repression

**Figure 1:**
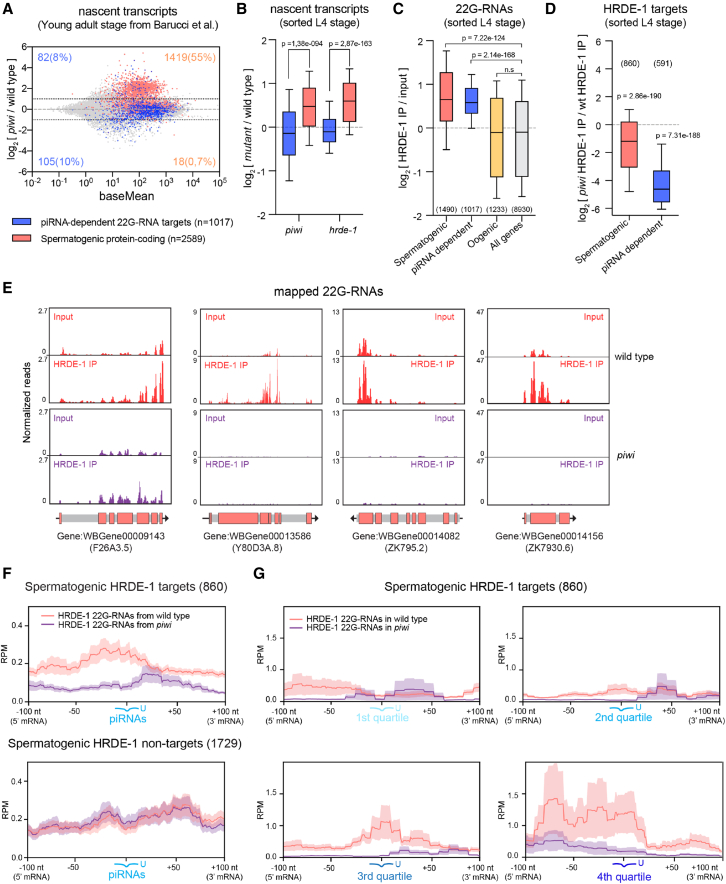
piRNAs target spermatogenic mRNAs to trigger their transcriptional silencing through HRDE-1 (A) MA-plot showing the log_2_ fold change of protein-coding nascent RNAs (GRO-seq) in *piwi* mutant versus wild-type young adult worms (data from Barucci et al., 2020). Dashed lines indicate 2-fold changes, and the colored numbers indicate the total number and proportion (in parentheses) of misregulated piRNA-dependent 22G-RNA targets (blue) or spermatogenic-enriched genes (light red) (2-fold changes; adjusted p < 0.05, Wald test). (B) Box plots showing the log_2_ fold change in nascent RNAs (GRO-seq) for piRNA-dependent 22G-RNA targets (blue) or spermatogenic-enriched genes (light red) in late L4 *piwi* and *hrde-1* mutant worms versus wild-type. (C) Boxplots showing the log_2_ fold change in 22G-RNAs (sRNA-seq) from HRDE-1 IPs compared with input for different categories of genes. The number of genes in each category is shown in parenthesis, considering only genes with >1 RPM in HRDE-1 IPs. (D) Boxplots showing the log_2_ fold change in 22G-RNAs (sRNA-seq) from *piwi* mutant versus wild-type HRDE-1 IPs for HRDE-1 targets. In (B–D), boxplots display median (line), first, and third quartiles (box), and 90^th^/10^th^ percentile values (whiskers), and two-tailed p values were calculated using Mann-Whitney-Wilcoxon tests. The number of genes is shown in parenthesis. (E) Genomic view of spermatogenic piRNA target genes. Panels show normalized 22G-RNA reads (reads per million, RPM) from total RNA (input) and HRDE-1 IPs in wild-type (red) and *piwi* mutants (purple). Colored boxes represent coding sequences, and gray boxes correspond to non-coding sequences (introns, UTRs). (F) RPM density of 22G-RNAs from HRDE-1 IPs in a 200-nt window around predicted piRNA targeting sites in wild-type (light red) or *piwi* (purple) mutants. Spermatogenic HRDE-1 targets and non-targets were analyzed separately. (G) RPM density of 22G-RNAs from HRDE-1 IPs around predicted piRNA-targeting sites on HRDE-1 spermatogenic targets. Data were analyzed based on piRNA expression levels, from least (1^st^ quartile) to most (4^th^ quartile) abundant.

To investigate genome-wide signatures of piRNA-mediated transcriptional silencing, we examined published global run-on sequencing (GRO-seq) data from young adult wild-type and *piwi* mutant hermaphrodites (Barucci et al., 2020), which lack piRNAs. When we looked at protein-coding genes corresponding to previously defined piRNA-dependent 22G-RNA targets—a category inferred based on global loss of total 22G-RNAs upon *piwi* mutation (Barucci et al., 2020)—we found that only 18% showed altered transcription in *piwi* mutants (Figure 1A). In contrast, 55% (1,419) of spermatogenic protein-coding genes (Ortiz et al., 2014) had increased nascent transcription in *piwi* mutants compared with wild-type (>2-fold; adjusted p < 0.05; Figure 1A). Spermatogenesis in the *C. elegans* hermaphrodite germline occurs during the L4 stage, preceding oogenesis that starts in young adult germlines (Figure S1A). To rule out the possibility that the upregulation of spermatogenic genes could reflect developmental differences between wild-type and mutant populations at the young adult stage, we performed GRO-seq in synchronized and sorted (see STAR Methods) wild-type and *piwi* mutant worms undergoing spermatogenesis (Figure S1A). We confirmed that, in contrast to genes corresponding to previously identified piRNA-dependent 22G-RNA targets (Barucci et al., 2020), spermatogenic genes were significantly upregulated in *piwi* mutant animals also during spermatogenesis (Figure 1B). Furthermore, a similar genome-wide signature was observed in animals mutant for the nuclear Argonaute HRDE-1, a downstream nuclear component of the piRNA pathway that binds 22G-RNAs and promotes RNAi- and piRNA-induced transcriptional gene silencing (Bagijn et al., 2012; Buckley et al., 2012) (Figures 1B and S1B). From these observations, we hypothesized that piRNAs directly silence the transcription of spermatogenesis genes through the production of 22G-RNAs loaded into HRDE-1.

To explore this possibility, we identified 22G-RNAs bound to HRDE-1::GFP in synchronized wild-type hermaphrodites undergoing spermatogenesis. We found enrichment of 22G-RNAs in HRDE-1::GFP immunoprecipitates (IPs) from previously defined piRNA-dependent 22G-RNA targets (Barucci et al., 2020) and spermatogenic-enriched mRNAs (Ortiz et al., 2014) (Figures 1C and 1E). A large fraction of these HRDE-1 spermatogenic targets is expressed specifically in sperm and male tissue (Figures S1C and S1D), suggesting that HRDE-1 can directly target and repress spermatogenic transcription. In contrast, we found no enrichment of 22G-RNAs from oogenic-enriched mRNAs (Ortiz et al., 2014) in HRDE-1 IPs (Figure 1C). To verify that piRNAs trigger the production and loading of spermatogenic 22G-RNAs into HRDE-1, we first confirmed that the HRDE-1-enriched 22G-RNAs were significantly depleted in *piwi* mutant animals (Figures 1D and 1E). This result indicates that a previously unappreciated subset of piRNA-dependent 22G-RNAs is generated from spermatogenic transcripts and loaded into HRDE-1. Next, we tested whether the production of 22G-RNAs loaded in HRDE-1 is directly triggered by piRNA-targeting events. To do so, we first identified predicted piRNA target sites along the *C. elegans* transcriptome by applying stringent matching criteria (Zhang et al., 2018) (see STAR Methods). Then, we analyzed the distribution of 22G-RNAs from HRDE-1 IPs mapping on a 200-nucleotide (nt) window centered around the identified piRNA-targeting sites. Our analysis showed the enrichment of 22G-RNAs toward the 5′ upstream piRNA-targeting region of spermatogenic HRDE-1 targets (Figure 1F). Moreover, the observed enrichment of 22G-RNAs was lost in the absence of piRNAs (Figure 1F). We also asked whether the piRNA-dependent synthesis of 22G-RNAs on spermatogenic targets correlated with piRNA expression levels and found higher levels of 22G-RNAs in the 5′ upstream region of the mRNAs containing target sites from highly expressed piRNAs (Figure 1G).

Altogether, these results indicate that piRNAs and PIWI directly trigger the production of spermatogenic 22G-RNAs loaded into HRDE-1 to promote the transcriptional repression of spermatogenic genes during spermatogenesis.

### The nuclear localization of HRDE-1 is exclusively dependent on piRNA signaling during spermatogenesis

**Figure 2:**
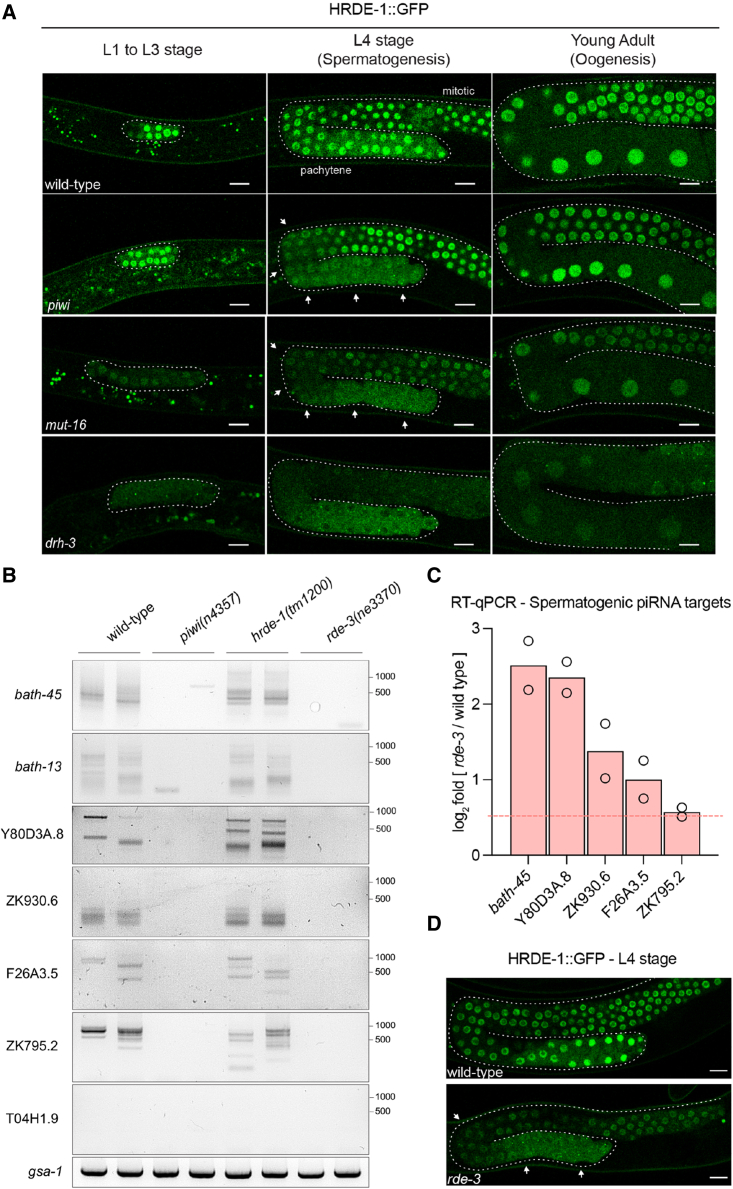
piRNA-dependent 22G-RNAs prime HRDE-1 nuclear localization during spermatogenesis (A) Panels showing a single confocal plane of live animal germlines expressing an HRDE-1::GFP reporter at the indicated developmental time points and genetic backgrounds. Arrows indicate pachytene-specific loss of nuclear HRDE-1 enrichment. Scale bars, 10 μm. (B) Gene-specific pUG assay (Shukla et al., 2020) (see STAR Methods) on the indicated mRNAs and genetic backgrounds. Results from two independent biological replicates. T04H1.9 is a non pUGylated mRNA, and *gsa-1* contains a pUG stretch genetically encoded in its 3′ UTR used as a loading control. (C) Expression levels of the indicated mRNAs in sorted L4 *rde-3(ne3370)* mutant worms by RT-qPCR. mRNA levels were normalized to *act-3*. Bars show the average levels from two biological replicates. (D) Panels show a single confocal plane of live wild-type and *rde-3(ne3370)* germlines expressing an HRDE-1::GFP reporter during spermatogenesis.

Because PIWI and piRNA signaling have been previously shown to restrict the subcellular localization of downstream cytoplasmic WAGO effectors (Barucci et al., 2020), we tracked HRDE-1::GFP localization during germline development, confirming the nuclear enrichment of HRDE-1 in all wild-type germ cells at all developmental stages (Figure 2A), as previously observed (Buckley et al., 2012). Strikingly, in the *piwi* mutant, we observed a fully penetrant loss of HRDE-1::GFP nuclear localization in pachytene nuclei undergoing spermatogenesis (Figure 2A, arrows). To further investigate whether 22G-RNAs regulate the nuclear localization of HRDE-1, we examined the *mut-16* mutant, which is deficient in the biogenesis of the 22G-RNAs required for piRNA-mediated silencing (Bagijn et al., 2012; Zhang et al., 2011), and a catalytic mutant of the Dicer-related helicase 3 (DRH-3), which abrogates the biogenesis of all 22G-RNAs (Gu et al., 2009). Our results show that whether *mut-16* animals phenocopied the fully penetrant loss of HRDE-1 in pachytene nuclei observed in *piwi* mutants (Figure 2A), *drh-3* mutant animals displayed impaired nuclear localization of HRDE-1 in the whole germline during all stages of development tested (Figure 2A). Therefore, the nuclear localization of HRDE-1 is driven by the loading of distinct populations of piRNA-dependent and -independent 22G-RNAs. In this context, piRNA signaling seems to be responsible for the nuclear function of HRDE-1 during spermatogenesis.

### piRNA targeting triggers the pUGylation of spermatogenic mRNAs

During RNA interference, the ribonucleotidyltransferase RDE-3 adds poly(UG) tails (pUGylation) to the 3′ end of cleaved mRNA fragments targeted for silencing, acting as a recruiting signal for RdRPs to promote the production of secondary 22G-RNAs (Shukla et al., 2020). Furthermore, some endogenous transcripts targeted by piRNAs are also subjected to pUGylation by RDE-3 in a PIWI-dependent manner (Shukla et al., 2021), suggesting that piRNA targeting triggers mRNA pUGylation. We detected PIWI-dependent pUGylated mRNA fragments in wild-type worms on spermatogenic piRNA targets (Figure 2B), similar to what has been previously shown for genes downregulated by piRNAs such as *bath-13* or *bath-45* (Shukla et al., 2021) (Figure 2B). These pUGylated RNAs were still detected in *hrde-1* mutants (Figure 2B), consistent with HRDE-1 functioning downstream of 22G-RNA synthesis but were undetectable in the *rde-3* mutant (Figure 2B), suggesting that RDE-3 pUGylates spermatogenic piRNA targets. To further implicate RDE-3 in piRNA-dependent spermatogenic transcriptional silencing, we show that in the *rde-3* mutant, spermatogenic piRNA targets are upregulated (Figure 2C) concomitantly with the loss of HRDE-1::GFP enrichment in the pachytene nuclei of *rde-3* mutant germlines during spermatogenesis (Figure 2D). These results are consistent with the role of piRNAs in initiating a transcriptional silencing of spermatogenic genes that requires the coordinated pUGylation of the spermatogenic mRNA targets, the synthesis of 22G-RNAs, and the nuclear localization of HRDE-1.

### PIWI is required for the incorporation of *Mutator* foci into P granules during spermatogenesis

**Figure 3:**
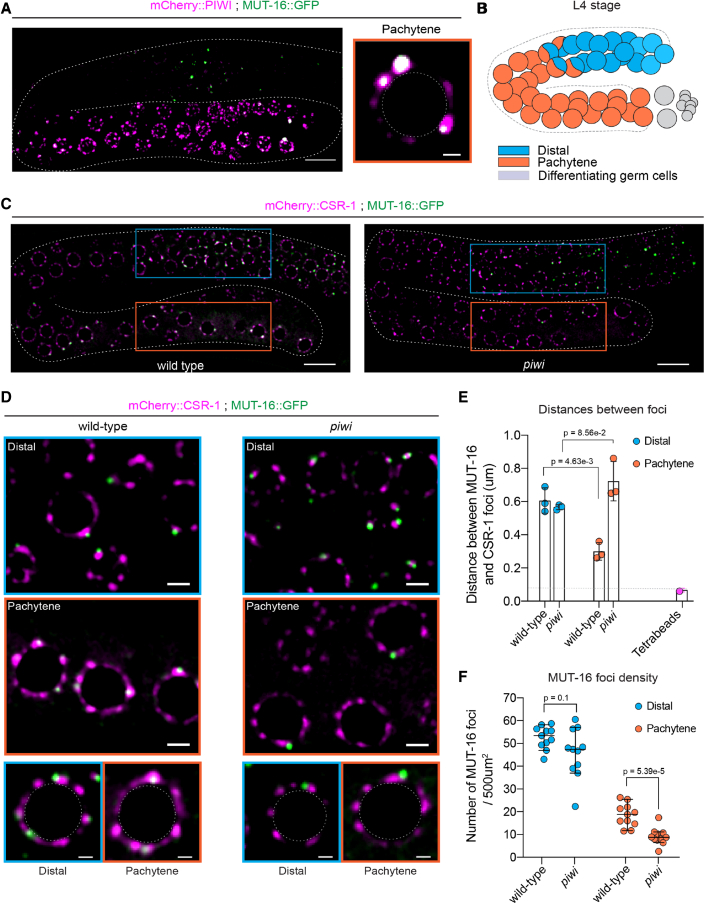
Spatiotemporal dynamics of *Mutator* foci and P granules during spermatogenesis (A) (Left) Panel showing a confocal z stack of germline surfaces from live animals expressing the indicated fluorescent proteins during the L4 stage. Scale bar, 10 μm. (Right) Fluorescent micrograph of a pachytene germ cell nucleus from animals expressing the indicated fluorescent proteins. Scale bar, 1 μm. (B) Schematic representation of the distal and pachytene regions of an L4 stage germline. Colored circles are germ cell nuclei. (C) Panels show a single confocal plane of live germlines expressing mCherry::CSR-1 and MUT-16::GFP in wild-type and *piwi* mutant animals during the L4 stage. Rectangular highlight germ cells in the germline’s distal (blue) and pachytene (orange) regions. Scale bars, 10 μm. (D) Distal and pachytene germ cells of animals expressing GFP::MUT-16 and mCherry::CSR-1 in wild-type and *piwi* mutant animals. Scale bar, 2 μm. Examples of individual germ cell nuclei are shown at the bottom. Scale bar, 1 μm. (E) Distance between the centers of MUT-16::GFP and mCherry::CSR-1 condensates. The bars indicate the mean value, and error bars indicate the standard deviation of 10 granules measured in 3 animals (n = 30 total). The last column shows the chromatic shift measured for tetraspeck beads (n = 30). Two-tailed p values were calculated using an unpaired t test. (F) MUT-16 foci density measured in different regions of live germlines of the indicated genotypes. The bar indicates the median value, and error bars indicate the 95% confidence interval (CI) of the number of MUT-16 foci measured in 10 individual germlines.

PIWI and its downstream components are expressed during germline development. Still, they are enriched into different biomolecular condensates in the perinuclear nuage of germ cells: while PIWI localizes to P granules, small RNA biogenesis factors such as MUT-16 and RDE-3 concentrate to *Mutator* foci (Phillips et al., 2012). We followed the subcellular localization of piRNA pathway components during spermatogenesis to discern the spatiotemporal specificity of nuclear piRNA-dependent signaling. In parallel, we studied the localization of CSR-1, a germline AGO protein highly enriched in P granules (Claycomb et al., 2009) and also present in Z granules (Charlesworth et al., 2021), another type of biomolecular condensate part of the *C. elegans* nuage (Wan et al., 2018). We confirmed the co-localization of PIWI and CSR-1 with GLH-1, the *C. elegans* homolog of the DEAD-box RNA helicase Vasa, a core P granule component (Figure S2A). However, while CSR-1 was ubiquitously expressed in the germline tissue (Figure S2A), PIWI was almost exclusively expressed in the pachytene region (Figures 3A and S2A), where we previously observed the piRNA-dependent loss of nuclear HRDE-1 signal. Moreover, MUT-16, a core component of *Mutator* foci, which are separated from P granules along with germline development (Uebel et al., 2020; Wan et al., 2018), co-localized with PIWI (Figure 3A), suggesting the specific incorporation of *Mutator* foci into P granules in the pachytene region (Figure 3B). To better characterize the dynamics between P granules and *Mutator* foci, we quantified the relative distances between endogenously tagged mCherry::CSR-1 and MUT-16::GFP in distal versus pachytene regions of the germline (Figures 3B and 3C). We found that the distance between the centers of the two condensates is reduced in the pachytene region compared with the distal germline, reflecting the integration of *Mutator* foci into P granule condensates (Figures 3D, 3E, and S2B). Moreover, *piwi* knockout mutants had a significantly reduced density of MUT-16 condensates surrounding pachytene germ cell nuclei (Figures 3F and S2C) and an impaired integration of *Mutator* foci into P granules in the pachytene region (Figures 3C–3E and S2C). In addition, IP-mass spec analysis revealed that PIWI interacts with MUT-16, RDE-3, and HRDE-1 (Barucci et al., 2020) (Figure S2D). Overall, these data show the dynamic and pachytene-specific incorporation of *Mutator* foci into P granules, which might depend on protein-protein interactions mediated by PIWI. This inclusion event correlates with the spatiotemporal specificity of piRNA-mediated nuclear HRDE-1 function during spermatogenesis.

### Spermatogenic piRNA targets are transiently expressed during germline development

**Figure 4:**
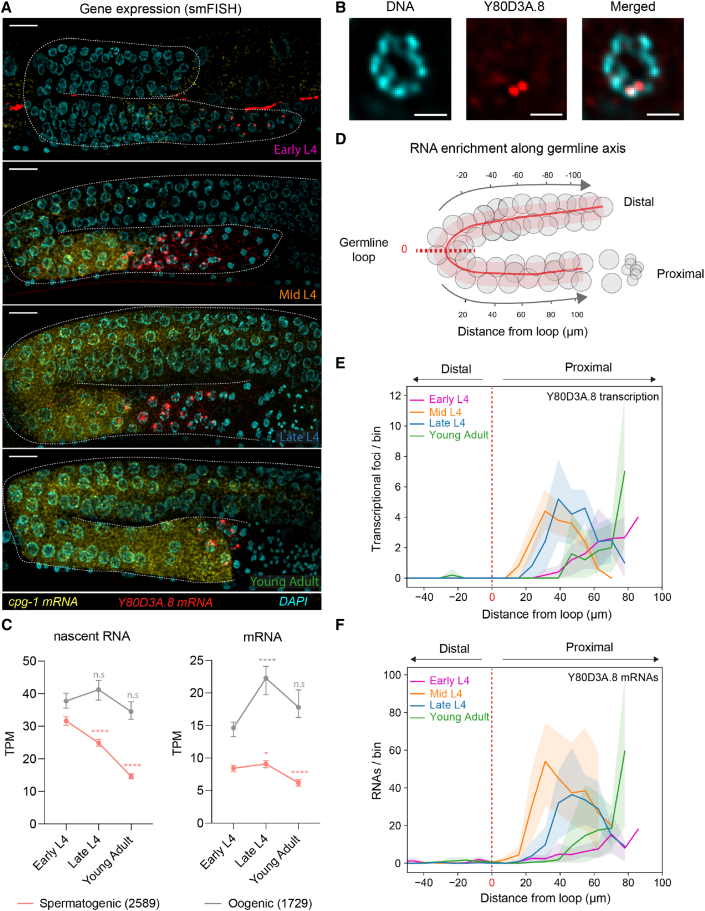
Expression dynamics of spermatogenic piRNA targets during germline development (A) smFISH of spermatogenic Y80D3A.8 mRNAs (red) and oogenic *cpg-1* mRNAs (yellow). DNA staining with DAPI (cyan). Scale bars, 10 μm. (B) Magnified view of a pachytene germ cell nucleus expressing Y80D3A.8 (red) and DAPI staining (cyan). Scale bars, 2 μm. (C) Detection of spermatogenic- and oogenic-enriched nascent RNAs (GRO-seq) and mRNAs (RNA-seq) from synchronized and sorted early L4, late L4, and young adult worms. Median levels and 95% confident interval of normalized read abundances in transcript per million (TPM) are shown. Two-tailed p values were calculated using Mann-Whitney-Wilcoxon tests (n.s: p > 0.5; ^∗^p < 0.5; ^∗∗∗∗^p < 0.0001); number of genes indicated in parenthesis. (D) Schematic representation of the pipeline for RNA spot detection along the germline axis from smFISH images. (E and F) Average and standard deviation (from n = 5 germlines) of spermatogenic Y80D3A.8 piRNA target transcriptional foci (E) and mRNAs (F) as a function of position in the germlines at the indicated developmental timepoints. RNA counts are attributed for each point along the axes, and data are binned for easier representation. The red dashed line indicates the position of the germline loop.

To explore the biological significance of the piRNA-mediated transcriptional silencing of spermatogenic genes, we followed the expression of two spermatogenic piRNA targets (Y80D3A.8 and ZK795.2) and two oogenic mRNAs (*cpg-1* and *puf-5*) during germline development by whole worm single molecule fluorescence *in situ* hybridization (smFISH). Moreover, exonic smFISH probes against the spermatogenic Y80D3A.8 piRNA target could also distinguish between nascent transcriptional foci and cytoplasmic mRNAs (Figures 4A, 4B, and S3A; STAR Methods). In agreement with spermatogenesis happening during the L4 stage in hermaphrodite germlines (Figure S1A), we detected expression of spermatogenic piRNA targets from the early L4 (pre-sperm formation) to young adult stages (mature sperm + oogenesis) in the most proximal region of the germline (Figures 4A and S4A). The quantification of nascent and mature Y80D3A.8 RNAs along the germline axis (Figure S3B and STAR Methods) showed a relatively stable domain of both transcription and mRNA expression in the proximal part of the gonad (Figures 4D–4F). Moreover, its expression was always maintained at a distance from the germline loop throughout all the stages of spermatogenic differentiation (Figures 4D–4F). Similar results were obtained when quantifying mRNAs of the ZK795.2 target (Figures S4A and S4B).

We also detected the expression of the oogenic *cpg-1* mRNA starting from the earliest phases of spermatogenesis (early and mid L4 stage) (Figure 4A), even though oogenesis starts only after L4, and spermatogenic and oogenic mRNAs localized to two mutually exclusive domains of the syncytial gonad (Figure S5A). These domains appear to divide meiotic germ cells into two transcriptionally distinct populations—oogenic and spermatogenic—from early L4 stages before any sign of mature gamete differentiation (Figure 4A). To extend our observations genome-wide, we performed GRO-seq and RNA-seq with sorted worm populations that were precisely staged at different steps of gamete differentiation: early L4 (pre-sperm formation), late L4 (mature sperm), and young adult (mature sperm + oogenesis) (see STAR Methods and Figures S4C and S4D). Our analyses confirmed that spermatogenic and oogenic transcription occur concomitantly from the early L4 stage (Figure 4C), and spermatogenic transcription progressively declines from early L4 to young adult stages due to sperm differentiation (Figure 4C).

The combination of genome-wide and smFISH data shows that spermatogenic gene expression is restricted in space and time in the hermaphrodite germline. It is transiently expressed during the L4 stage for approximately 10 h in pachytene germ cells at the most proximal region, concomitant with oogenic transcription in the immediately adjacent distal region, toward the germline loop.

### piRNAs repress spermatogenic transcription in pachytene nuclei undergoing spermatogenesis

**Figure 5:**
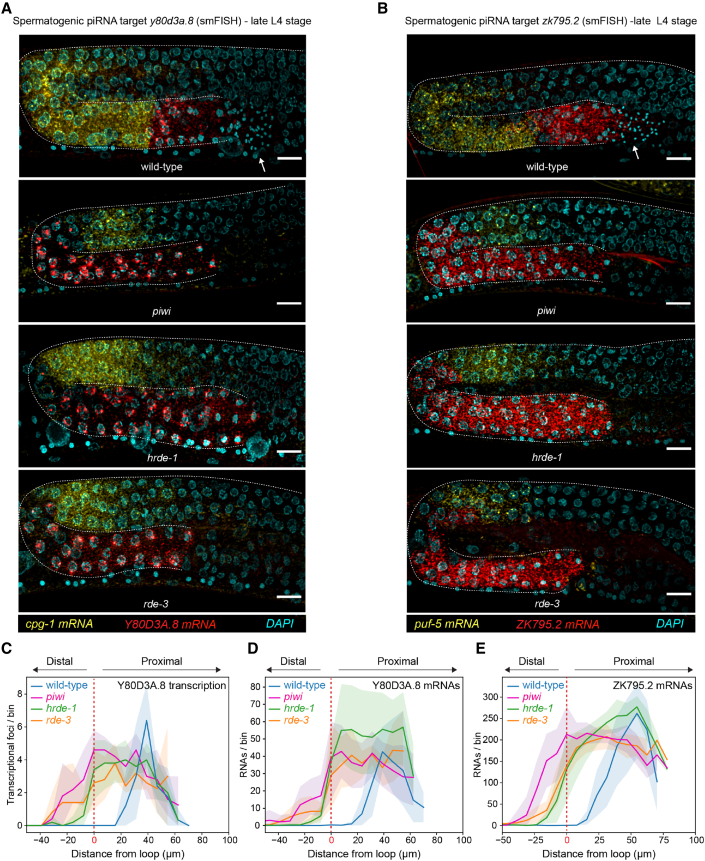
piRNA-mediated transcriptional silencing is required for spermatogenic differentiation (A and B) Late L4 smFISH of spermatogenic Y80D3A.4 (red) and oogenic *cpg-1* (yellow) mRNAs (A) or ZK795.2 (red) and *puf-5* (yellow) mRNAs (B). DNA staining with DAPI (cyan). Scale bars, 10 μm. (C–E) Average and standard deviation (from n = 5 germlines) of spermatogenic Y80D3A.8 piRNA target transcriptional foci (C) and mRNAs (D) or spermatogenic ZK795.2 piRNA target mRNAs (E) as a function of position in the late L4 germlines of the indicated genotypes. The red dashed line indicates the position of the germline loop.

Next, we characterized the expression of spermatogenic piRNA targets in different piRNA pathway mutants by smFISH. We observed an increased number of pachytene germ cells expressing the spermatogenic Y80D3A.8 and ZK795.2 mRNAs during the late L4 stage (Figures 5A, 5B, and S5A), consistent with the upregulation of spermatogenic genes in *piwi*, *hrde-1*, and *rde-3* mutants (Figures 1B and 2C). Furthermore, pachytene germ cells actively transcribing Y80D3A.8 were observed along the germline's proximal region and reached the germline loop (Figures 5B and S5A), invading the region where oogenic mRNAs usually accumulate in late L4 wild-type germlines. Consequently, the domain of oogenic expression appeared retracted in *piwi*, *hrde-1*, and *rde-3* mutants (Figures 5C and 5D). In wild-type germlines, the domain of spermatogenic transcription is restricted to a narrow group of pachytene germ cells at the proximal end and precedes the transition of germ cells to the condensation zone, a region associated with the global transcriptional repression and condensation of nuclear content (Shakes et al., 2009) necessary to ensure meiotic divisions and sperm maturation (Figure S5B). The increased number of germ cells transcribing sperm genes in piRNA mutants was also correlated with the absence of post-meiotic spermatogenic germ cells at the late L4 stage (Figures 5A, 5B, and S5B), suggesting that the defect in repressing spermatogenic gene transcription affects the dynamics of spermatogenic differentiation. Of note, these molecular phenotypes spatially coincide with the region where HRDE-1 loses nuclear enrichment in piRNA pathway mutants (Figures 2A and 2D).

We tracked HRDE-1, PIWI, and MUT-16 localization in wild-type and *piwi* mutant males to study whether these molecular phenotypes also occur during male spermatogenesis. We found that piRNAs were also required for the nuclear localization of HRDE-1 in the pachytene nuclei along with male germline development (Figure S6A). In addition, this is the region where we also observed the inclusion of MUT-16 foci within P granules containing PIWI (Figure S6B) and the repression of spermatogenic gene expression (Figure S6C). Overall, these results suggest that the repression of spermatogenic gene transcription by piRNAs during spermatogenesis is globally required for male gametogenic gene expression programs.

### Transcriptional repression mediated by piRNAs promotes germ cell differentiation and function

**Figure 6:**
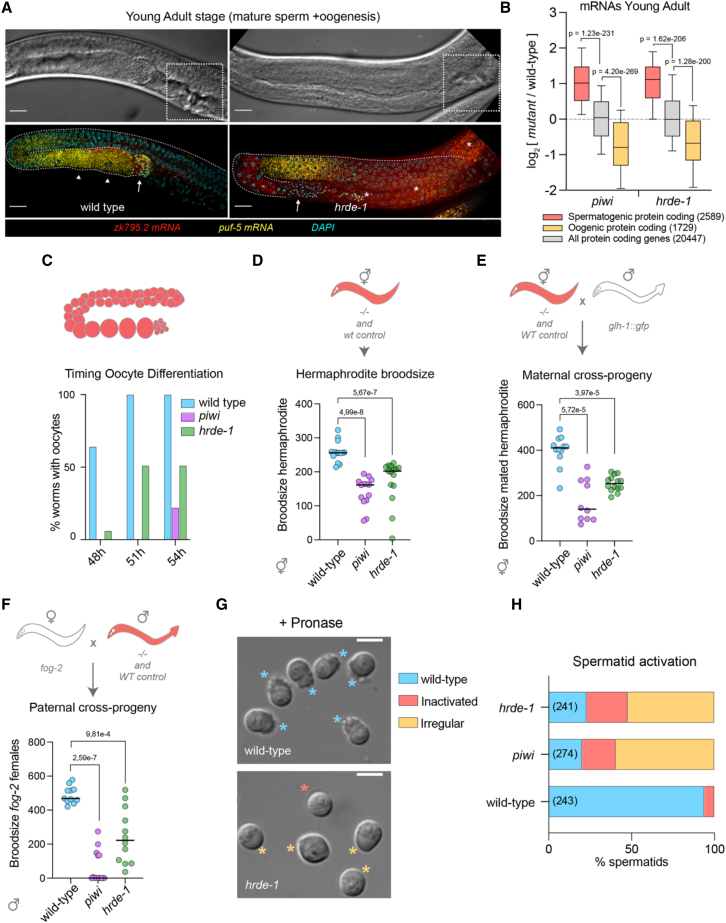
Transcriptional repression of spermatogenic genes by piRNAs is required for fertility (A) Expression of spermatogenic ZK795.2 piRNA target (red) and oogenic *puf-5* mRNAs (yellow) by smFISH in young adult *hrde-1* mutant or wild-type germlines. DNA staining with DAPI (cyan). Arrows indicate mature sperm and arrowhead oocytes. Asterisks highlight regions with high background autofluorescence. Scale bars, 15 μm. Upper panels: DIC images. White dashed squares highlight the presence of a fully formed vulva in both wild-type and mutant worms. (B) Boxplots showing the log_2_ fold change in mRNAs (RNA-seq) for spermatogenic- (light red), oogenic-enriched (yellow), and all genes (gray) in synchronized young adult *piwi* and *hrde-1* mutant worms versus wild-type (data from Barucci et al., 2020). Boxplots display median (line), first, and third quartiles (box), and 90^th^/10^th^ percentile values (whiskers). Two-tailed p values were calculated using Mann-Whitney-Wilcoxon tests. The number of genes is reported in parenthesis. (C) The presence of oocytes was scored from synchronized young adult individuals of the indicated genotypes and at different time points (n = 50 worms scored per time point and genotype). (D) Brood size of wild-type, *piwi*, and *hrde-1* mutant hermaphrodites. Data points correspond to the number of alive F1 larvae from individual worms. Bars indicate the median brood size value for each population. Two-tailed p values were calculated using Mann-Whitney-Wilcoxon test. (E) *hrde-1* and *piwi* mutant hermaphrodites show fertility defects when mated with wild-type males. Data points correspond to the number of alive F1 larvae from individual worms. Bars indicate the median brood size value for each population. Two-tailed p values were calculated using Mann-Whitney-Wilcoxon test. (F) *hrde-1* and *piwi* mutant males show fertility defects when mated with fog-2 females. Data points correspond to the number of alive F1 larvae from individual worms. Bars indicate the median brood size value for each population. Two-tailed p values were calculated using Mann-Whitney-Wilcoxon test. (G) Representative images of the *in vitro* sperm activation assay from wild-type, *piwi*, and *hrde-1* mutant males. Pronase-treated mutant spermatids exhibit activation and morphological defects. Scale bars, 5 μm. (H) Percentage of activated, irregular, and inactivated spermatids from a sperm activation assay on males of the indicated genetic backgrounds. At least 10 adult male animals were dissected. A total number of spermatids scored is reported in parenthesis.

We then evaluated whether the observed alterations in gene expression programs impact gamete function and animal fertility. We noticed that the domain of spermatogenic gene expression was still present in germlines of *piwi* and *hrde-1* mutants at young adult stages (Figures 6A and S6D), where wild-type germlines are already starting oogenic differentiation and therefore do not transcribe spermatogenic genes (Figure 6A). RNA-seq in synchronized young adult *piwi* and *hrde-1* mutants confirmed a significant increase of spermatogenic mRNAs and reduced expression of oogenic mRNAs compared with wild-type worms (Figure 6B). Furthermore, due to the continuous expression of spermatogenic genes and delayed sperm differentiation in *piwi* and *hrde-1* mutants, the onset of oogenesis was also severely delayed (Figure 6C) and correlated with a reduced brood size phenotype (Figure 6D). To investigate whether the defect in repressing spermatogenic transcription impacts the number and/or quality of oocytes, we scored the number of maternal cross progenies in *piwi* and *hrde-1* mutant hermaphrodites after mating with wild-type males carrying a germline GLH::GFP marker. Both *piwi* and *hrde-1* mutant hermaphrodites showed a significantly reduced number of maternal cross-progeny (based on the presence of germline GFP expression in F1) compared with wild-type (Figure 6E). Moreover, to evaluate the impact of nuclear piRNA signaling on male fertility, we mated genetically induced *fog-2* females, unable to produce sperm, with wild-type males or *piwi* and *hrde-1* male mutants. Paternal cross-progeny from *fog-2* females crossed with *piwi* and *hrde-1* mutant males was significantly reduced compared with wild-type males (Figure 6F), suggesting that male fertility is affected in the absence of nuclear piRNAs signaling.

To investigate the cause of the reduced male fertility observed in *piwi* and *hrde-1* mutants, we examined sperm quality and function. The last step of spermatogenic differentiation in the male germline occurs after ejaculation, where immature spermatids undergo spermiogenesis and acquire a functional pseudopod required for sperm motility and fertilization (Smith, 2014). Therefore, we induced spermiogenesis *in vitro* by treating isolated spermatids with pronase (Shakes and Ward, 1989) and found that in the absence of PIWI or HRDE-1, differentiated spermatids fail to produce wild-type pseudopod structures (Figures 6G and 6H).

Altogether these results show that the repression of spermatogenic gene transcription by piRNAs and the downstream nuclear HRDE-1 is essential for sperm function and animal fertility.

### The CSR-1 pathway license spermatogenic gene expression during spermatogenesis

**Figure 7:**
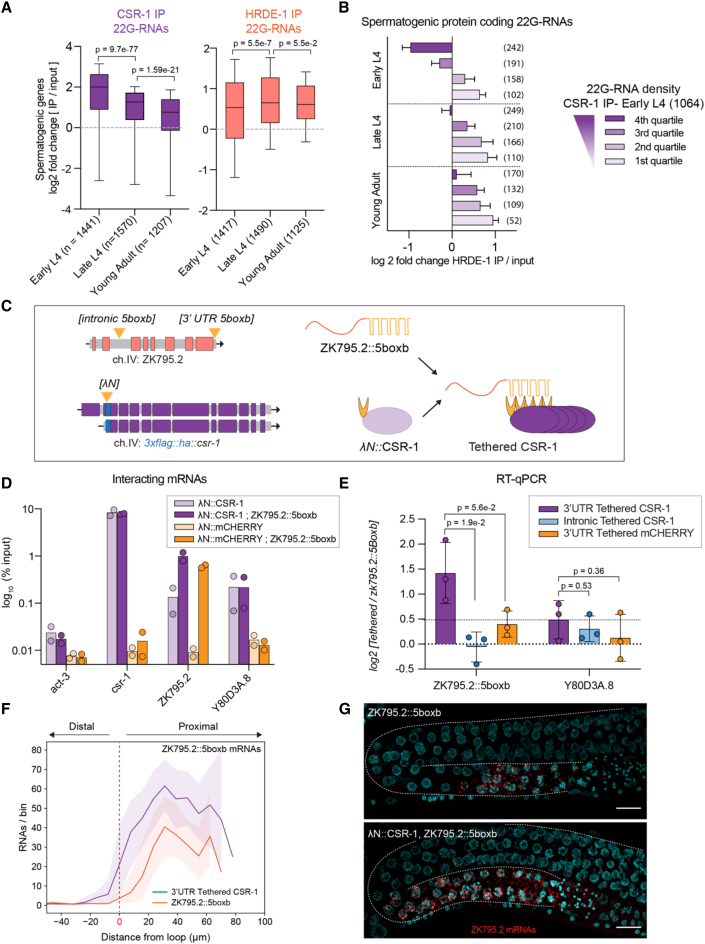
Tethering of AGO CSR-1 to a spermatogenic mRNA confers protection against piRNA targeting (A) Boxplots show the log_2_ fold change of spermatogenic 22G-RNAs (sRNA-seq) in HRDE-1 and CSR-1 IPs compared with input in wild-type animal populations at three developmental time points. Boxplots display median (line), first, and third quartiles (box), and 90^th^/10^th^ percentile values (whiskers). Two-tailed p values were calculated using Mann-Whitney-Wilcoxon tests; the number of genes is indicated in parenthesis. (B) Histograms show the log_2_ fold change of CSR-1 loaded 22G-RNAs (sRNA-seq) at early L4 in HRDE-1 IPs compared with input in wild-type animal populations at three developmental time points. Early L4 CSR-1 spermatogenic targets were ranked in quartiles of 22G-RNA density in CSR-1 IPs. The bars indicate the median, and error bars indicate a 95% confidence interval. Numbers in parentheses indicate the portion of CSR-1 targets analyzed in each category. (C) Diagram of the endogenous CSR-1 tethering assay. Colored boxes represent coding sequences, and gray boxes correspond to non-coding sequences (introns, UTRs). (D) RNA immunoprecipitation (RIP) experiments followed by RT-qPCR showing the log_10_ percentage of input for a known CSR-1 target (*csr-1*) (Singh et al., 2021), and two spermatogenic piRNA targets (ZK795.2 and Y80D3A.8) from λN::CSR-1 and λN::Cherry IPs at the indicated genetic backgrounds. *act-3* was used as a non-specific target gene. The bars indicate the mean value from n = 2 biologically independent experiments. (E) RT-qPCR log_2_ fold change of the spermatogenic piRNA targets ZK795.2 and Y80D3A.8 in late L4 sorted populations of λN::*csr-1;*ZK795.2::5boxb (tethered) worms compared with ZK795.2::5boxb control animals. The bar indicates the mean value, and error bars indicate the standard deviation. n = 3 biologically independent experiments. Statistical analysis was performed using two-tailed unpaired t tests. (F) Average and standard deviation (from n = 10 germlines) of ZK795.2::5boxb mRNAs as a function of position in the germlines of λN::*csr-1;*ZK795.2::5boxb (tethered) worms compared with ZK795.2::5boxb control animals. The red dashed line indicates the position of the germline loop. (G) Representative images of smFISH of the spermatogenic piRNA target ZK795.2 (red) in fixed late L4 germlines from the indicated genetic backgrounds. DNA staining with DAPI (cyan). Scale bars, 10 μm.

The robust spermatogenic gene transcription observed in the most proximal region of the germline suggested the existence of a protective signal counteracting piRNA-mediated silencing in this region. CSR-1 loads 22G-RNA antisense to the majority of germline-expressed genes (Claycomb et al., 2009) can target nascent RNAs to promote transcription (Cecere et al., 2014) and protect single-copy transgenes from piRNA-mediated silencing (Wedeles et al., 2013). To test whether CSR-1 targets and protects spermatogenic transcripts from the piRNA pathway, we examined the dynamics of 22G-RNA loading into CSR-1 and HRDE-1 during gamete differentiation. We combined our worm population sorting strategy with CSR-1 or HRDE-1 IPs, followed by small RNA sequencing. Given the expression of two isoforms of CSR-1 in the L4 stage (Charlesworth et al., 2021; Nguyen and Phillips, 2021), we verified the enrichment of both CSR-1 isoforms 22G-RNA targets in our sorted CSR-1 IPs (Figure S7A). The analysis of 22G-RNAs from sorted early L4 to young adult worms showed that oogenic 22G-RNAs were globally loaded into CSR-1 but not in HRDE-1 (Figure S7B). The spermatogenic 22G-RNAs were instead loaded in both CSR-1 and HRDE-1, although with slightly different dynamics (Figure 7A). Indeed, whereas the abundance of 22G-RNAs antisense to spermatogenic genes loaded into CSR-1 decreased over time following spermatogenic transcription (Figure 4C), the loading of spermatogenic 22G-RNA into HRDE-1 significantly increased from late L4. In addition, HRDE-1 preferentially loaded spermatogenic 22G-RNAs that were least abundant in or depleted from CSR-1 IPs (Figures 7B and S7C), suggesting that CSR-1 and HRDE-1 compete for the loading of spermatogenic 22G-RNAs.

The antagonistic relationship and dynamics of CSR-1 and HRDE-1 22G-RNA loading support a model where CSR-1 might preferentially bind and protect transcribed spermatogenic mRNAs and that the decreased CSR-1 mRNA interaction at later stages might favor piRNA targeting and silencing. CSR-1 post-transcriptionally regulates mRNA target levels through its catalytic activity (Gerson-Gurwitz et al., 2016; Singh et al., 2021), including those coding for germline AGOs and core P granule components (Figure S7D). As a result, *csr-1* mutants show an enlarged P granule phenotype (Figure S7E). In addition, *csr-1* knockout and catalytic dead mutant animals showed upregulated spermatogenic transcription in late L4 (Figure S7F), similar to piRNA pathway mutants (Figure 1B) and animals depleted of core P granule factors (Campbell and Updike, 2015). Based on these observations, we reasoned that the pleiotropic effects caused by *csr-1* mutations on germ granule integrity complicate the interpretation of the sequencing results using *csr-1* mutants.

To overcome this limitation, we adapted an *in vivo* tethering system (Wedeles et al., 2013) to determine how the continuous presence of endogenous CSR-1 on a single spermatogenic mRNA target affects its expression. The spermatogenic mRNA ZK795.2 accumulates HRDE-1 bound 22G-RNA mapping to the 3′ end region of the transcript (Figure 1E). We used CRISPR-Cas9 to tag the endogenous ZK795.2 transcript with five copies of the lambda phage *box b* RNA hairpin (ZK795.2::5boxb) in one intron or the 3′ UTR region and tagged the two isoforms of CSR-1 with the lambda phage N anti-termination protein fragment (λN::CSR-1), which is recruited to the *box b* hairpins (*Tethered*) (Figure 7C). The continuous tethering of λN::CSR-1 to ZK795.2::5boxb mRNAs caused a 10-fold increase in their interaction compared with control IPs (Figure 7D), similar to the tethering of a control λN::Cherry protein expressed under the strong and ubiquitous *hsp-90* promoter (Figures 7D and S7G). However, only the 3′ UTR tethering of λN::CSR-1 resulted in increased levels of ZK795.2::5boxb expression (Figure 7E). These results show that the stabilization of ZK795.2::5boxb mRNA levels is specific to CSR-1 tethering to the mature RNA, suggesting that the competition between CSR-1 and PIWI possibly occurs in the P granules. We also observed the binding of λN::CSR-1 and not λN::Cherry to endogenous CSR-1 targets and spermatogenic piRNA targets, confirming that CSR-1 can directly bind spermatogenic piRNA targets (Figure 7D). Finally, smFISH of ZK795.2::5boxb tethered to λN::CSR-1 revealed its expression in the pachytene region toward the germline loop (Figures 7F and 7G), reproducing the molecular phenotype observed in piRNA pathway mutants.

## Discussion

PIWI-interacting small RNAs are an example of flexibility and specificity in gene regulation. In worms, the mechanisms of piRNA-mediated repression of sequences encoded by foreign DNA such as single-copy transgenes or transposons seem to be operating, at least partially, on endogenous germline-expressed genes. For example, among the over 15,000 distinct *C. elegans* piRNA sequences, only a few individual cases have been shown to regulate post-transcriptionally—but not silence—the levels of endogenous mRNAs (Shen et al., 2018; Tang et al., 2018), suggesting that piRNAs can also fine-tune gene expression.

Here, by studying the function of small RNA pathways in the context of *C. elegans* hermaphrodite germline development, we report an unprecedented role of piRNAs in the global regulation of endogenous transcriptional programs. Using HRDE-1-bound 22G-RNAs as a readout of effective nuclear piRNA silencing, we show that by directly guiding the transcriptional repression of hundreds of spermatogenic protein-coding genes during spermatogenesis, piRNAs promote fertility and ensure spermatogenic differentiation and function.

piRNA-mediated silencing in *C. elegans* does not necessarily rely on perfect antisense complementarity (Bagijn et al., 2012; Lee et al., 2012; Shen et al., 2018; Zhang et al., 2018). For this reason, a common strategy used to infer piRNA targets has been to look for the reduction of global 22G-RNA levels concomitant to the upregulation of respective mRNA targets upon *piwi* mutation (Barucci et al., 2020; Zhang et al., 2018). In this context, the upregulation of spermatogenic genes in piRNA pathway mutants is not accompanied by a global decrease of respective antisense 22G-RNAs (Barucci et al., 2020; Reed et al., 2020), and this is why it has been previously considered to be an indirect effect (Reed et al., 2020). The similar upregulation of spermatogenic transcription in mutants of the nuclear AGO HRDE-1, a well-characterized downstream effector of the piRNA pathway, prompted us to explore the direct involvement of piRNAs in transcriptional regulation of sperm genes. The fact that HRDE-1 is not the only AGO loading 22G-RNAs and targeting spermatogenic genes might explain why the absence of piRNAs does not cause a global loss of spermatogenic 22G-RNAs. Indeed, the CSR-1 pathway also targets spermatogenic mRNAs (Charlesworth et al., 2021; Nguyen and Phillips, 2021). Thus, only by filtering 22G-RNA populations loaded into HRDE-1 in the presence or absence of piRNAs we were able to find a specific subset of 22G-RNAs following the established criteria to define putative direct piRNA regulation.

Surprisingly, the transcription of previously defined piRNA targets (Barucci et al., 2020) remained unchanged in *piwi* and *hrde-1* mutants, despite observing a significant loss of loaded 22G-RNAs from HRDE-1 IPs. Our interpretation is that piRNAs and HRDE-1 are only required for initiating the silencing of these targets, which are continuously maintained silenced by chromatin regulators. In fact, only minor changes in piRNA target expression were previously observed in piRNA mutants (Barucci et al., 2020), and the same effect is observed with the silencing of single-copy transgenes, which is initiated by piRNAs and can be maintained by nuclear factors in the absence of piRNAs (Ashe et al., 2012; Lee et al., 2012). However, further work is required to understand the mechanisms and dynamics of repression on different classes of piRNA targets, including spermatogenic genes.

**Figure 8:**
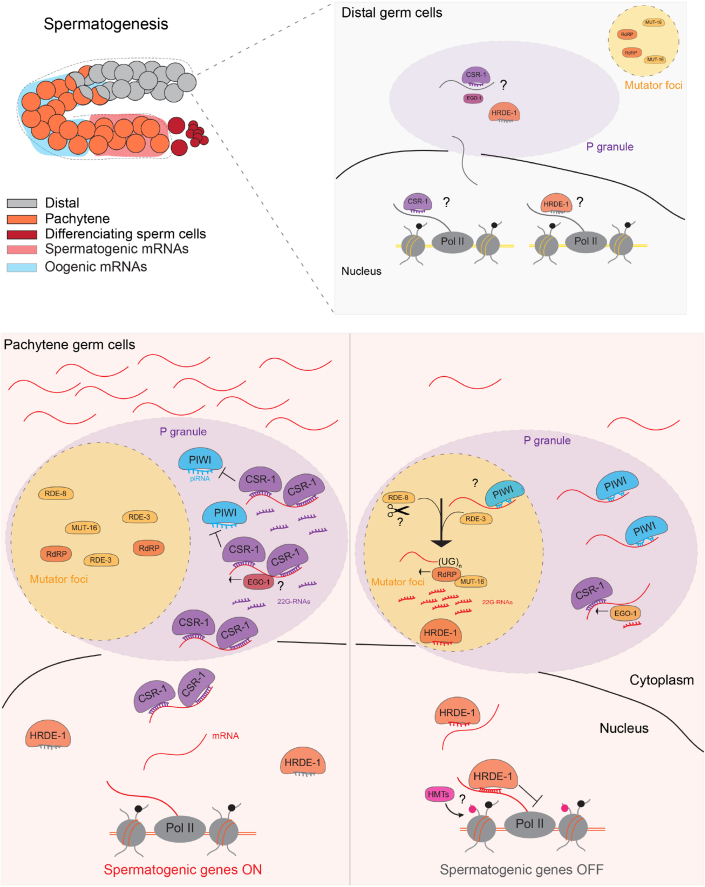
Model for the regulation of spermatogenic transcription by piRNAs During spermatogenesis, P granules and *Mutator* foci are two distinct condensates in the nuage of distal germ cells. In meiotic germ cells transiting the pachytene region, the expression and localization of PIWI to P granules is associated with the incorporation of the *Mutator* foci into P granules. This inclusion concentrates upstream and downstream factors required for the biogenesis of piRNA-dependent 22G-RNAs and nuclear piRNA signaling around transcripts exiting from the nuclear pore. CSR-1 targeting provides temporal protection from piRNA silencing in the P granules, licensing spermatogenic transcripts in the proximal region of the germline. As spermatogenic proceeds, loading of 22G-RNA antisense to spermatogenic genes is reduced in CSR-1, favoring piRNA targeting of spermatogenic transcripts and the synthesis and loading of 22G-RNAs in HRDE-1. The RdRP synthesis of piRNA-dependent 22G-RNAs on spermatogenic mRNAs requires the addition of polyUG stretches by RDE-3 on mRNA fragments, possibly cleaved RDE-8. The transcriptional silencing of spermatogenic genes by piRNAs promotes the correct meiotic differentiation of spermatogenic germ cells and confers temporal precision to the developmental switch from sperm to oocyte production.

We provide evidence that the transcriptional silencing by piRNAs requires the activities of proteins localizing to distinct phase-separated condensates, the P granules, and the *Mutator* foci. Whereas the targeting of mRNAs by piRNAs occurs in the P granules, the components required to produce piRNA-dependent 22G-RNAs localize to the *Mutator* foci. How the piRNA targeting and the synthesis and loading of 22G-RNAs onto downstream Argonaute proteins are coordinated in these two distinct condensates to trigger a transcriptional silencing signal is currently unknown. We have shown that PIWI directs the incorporation of *Mutator* foci into P granule condensates during spermatogenesis. We speculate that the reorganization of these two distinct liquid-like condensates is a mechanism used by piRNAs to trigger the global transcriptional silencing of spermatogenic genes efficiently. By concentrating upstream and downstream piRNA pathway components around spermatogenic mRNAs accumulating in P granules, this fusion event facilitates the RDE-3-mediated pUGylation at the sites of piRNA targeting and the synthesis of 22G-RNAs by RdRPs on pUGylated mRNA fragments. This signaling, in turn, allows loading of these 22G-RNAs onto the nuclear AGO HRDE-1, driving its nuclear localization to mediate repression of spermatogenic transcription in pachytene nuclei (Figure 8).

Which PIWI-dependent mechanisms drive the incorporation of *Mutator* foci into P granules? In *Drosophila*, piRNA loading into the PIWI protein Aubergine causes a conformational change leading to specific post-translational modifications. This promotes the interaction with scaffolding factors coordinating nuage assembly, piRNA amplification, and silencing (Huang et al., 2021). We speculate that similar mechanisms might regulate the observed PIWI-dependent incorporation of *Mutator* foci into P granules during *C. elegans* spermatogenesis. For example, mechanisms associated with the developmentally regulated transcription of different sets of piRNA sequences in the *C. elegans* germline (Choi et al., 2021) could direct the specific enrichment of PIWI and/or the reorganization of nuage condensates in the pachytene region during spermatogenesis. However, we cannot exclude the possibility that the incorporation of *Mutator foci* into PIWI-enriched P granules is a consequence of increased piRNA targeting and signaling on spermatogenic mRNAs accumulating in P granules during spermatogenesis. Whether similar or alternative unknown mechanisms might be used to promote silencing of specific subsets of mRNAs, such as RNAs derived from single-copy transgenes and TEs in the germline, requires further investigation.

In theory, the specific enrichment of PIWI and *Mutator foci* in the P granules of germ cells undergoing spermatogenesis should allow the robust piRNA-mediated silencing of most spermatogenic mRNAs. However, we show that piRNA silencing of spermatogenic mRNAs is restricted by the counteractive activity of the CSR-1 pathway. As a result, the overlap between opposing activities of PIWI and CSR-1 on sperm mRNAs in the hermaphrodite germline contributes to refining the transient expression of spermatogenic genetic programs and confers temporal precision to the developmental switch from sperm to oocyte production (Figure 8). Based on our tethering experiments, we propose that the competition between PIWI (silencing) and CSR-1 (anti-silencing) occurs on P granule-localized spermatogenic mRNAs. However, we cannot exclude that in some other cases, CSR-1 can also protect nuclear transcripts from HRDE-1 silencing activity.

The integrity of the *C. elegans* nuage is required to protect germ cell fate, a function that has been associated in part with the post-transcriptional regulation of somatic transcripts exiting the nucleus (Knutson et al., 2017; Updike et al., 2014). Our results show that changes in the composition of perinuclear liquid-like condensates present in the nuage correlate with changes in small-RNA-mediated signaling and nuclear gene activity. These observations strongly suggest that the *C. elegans* nuage might also facilitate global transcriptional programming during germ cell differentiation.

Our work reveals that piRNAs can function beyond the repression of invading genetic elements. In mammals, non-transposon-derived pachytene piRNAs are the most abundant class of piRNAs expressed during spermatogenesis (Aravin et al., 2006) and are required for sperm function (Wu et al., 2020). Furthermore, different studies have shown that a fraction of pachytene piRNAs can target meiotic protein-coding genes for post-transcriptional regulation (Goh et al., 2015; Gou et al., 2014; Vourekas et al., 2012; Wu et al., 2020; Zhang et al., 2015). However, the targeting rules and the mechanism used to regulate these mRNAs are unclear. In addition, the function and targets of a large fraction of pachytene piRNAs remain uncharacterized (Ozata et al., 2019). Overall, these studies show that piRNA functions can be co-opted to regulate endogenous genes during spermatogenesis, a strategy that appears to be evolutionary maintained despite the lack of piRNA sequence conservation across species. The function of piRNAs as global repressors of the *C. elegans* spermatogenic transcriptional program described here shows that the regulatory influence of piRNAs on endogenous genes is far from being residual. Thus, our study, together with previous work, contributes to expanding the notion that piRNAs function as a cellular immune system and act as highly versatile regulators of endogenous gene expression in animals.

### Limitation of the study

In this study, we have tethered CSR-1 to spermatogenic mRNAs to demonstrate the capacity of CSR-1 to prevent piRNA targeting and silencing. This is because *csr-1* mutations cause many pleiotropic effects, which also affect germ granule integrity. However, we could not demonstrate that the increased expression of a spermatogenic target tethered with CSR-1 was caused by impaired piRNA targeting. It is, in fact, possible that the tethering of CSR-1 helps stabilize the tethered mRNA despite piRNA targeting. We have also shown an upregulation of spermatogenic transcription in piRNA pathway mutants, but we have not evaluated at which generation these changes occur. This might be an important aspect to consider, given that gene expression changes in piRNA mutants are revealed only upon multiple generations (Barucci et al., 2020).

## STAR★Methods

### Key resources table

**Table:** 

REAGENT or RESOURCE	SOURCE	IDENTIFIER
**Antibodies**		

Anti-FLAG M2 Magnetic Agarose Beads	Sigma-Aldrich	Cat# M8823; RRID: AB_2637089
GFP-Trap Agarose Beads	ChromoTek	Cat# gta-20; RRID: AB_2631357

Bacterial and virus strains		
*Escherichia coli* strain : OP50	Caenorhabditis elegans Center	OP50

**Chemicals, peptides, and recombinant proteins**		

Cas9-NLS protein TrueCut V2 (5mg/mL)	Invitrogen	Cat#A36498
TRI Reagent	Invitrogen	Cat# AM9738
Turbo Dnase	Invitrogen	Cat#AM2238
HALT Protease Inhibitors	Thermo Fisher Scientific	Cat#78425
RiboLock RNAse Inhibitor	Thermo Fisher Scientific	Cat#EO0381
75 mM Bio-11-UTP	Invitrogen	Cat#10269614
T4 Polynucleotide Kinase	New England Biolabs	Cat#M0201L
DreamTaq DNA polymerase	Thermo Fisher Scientific	Cat#11883813
pronase E	Sigma	Cat#7433-2

**Critical commercial assays**		

Qubit Fluorometer High Sensitivity dsDNA assay kit	Thermo Fisher Scientific	Cat#Q32851
NextSeq 500/550 High Output v2 kit 75 cycles	Illumina	Cat# FC-404-2005
NEBNext Ultra II Directional RNA Library Prep Kit for Illumina	New England Biolabs	Cat#E7760S
SuperScript IV Reverse Transcriptase	Thermo Fisher Scientific	Cat#18090010
NEBNext Ultra II Q5 Master Mix	New England Biolabs	Cat#M0544L
M-MLV reverse transcriptase	Invitrogen	Cat#28025013

**Deposited data**		

All sequencing data	This study	GSE157319
Original polyUG PCR gel images	This study	DOI:10.17632/tnmxzg8wjj.1

**Experimental models: Organisms/strains**		

*C. elegans strain: wild isolate (Bristol)*	CGC	N2
*C. elegans strain: piwi(n4357) I*	CGC	SX922
*C. elegans strain: hrde-1(tm1200) II*	CGC	FX1200
*C. elegans strain: rde-3(ne3370)*	CGC	WM286
*C. elegans strain: ggSi1[hrde-1p::3xflag::gfp::hrde-1] II*	S. Kennedy lab	YY584
*C. elegans strain: piwi(gcp034[STOP]) I; ggSi1[hrde-1p::3xflag::gfp::hrde-1] II*	This study	MHE109
*C. elegans strain: piwi(gcp035[STOP]) I; ggSi1[hrde-1p::3xflag::gfp::hrde-1] II*	This study	MHE110
*C. elegans strain: drh-3(ne4253) I; ggSi1[hrde-1p::3xflag::gfp::hrde-1] II*	This study	MHE138
*C. elegans strain: mut-16(pk710) I; ggSi1[hrde-1p::3xflag::gfp::hrde-1] II*	This study	MHE141
*C. elegans strain: rde-3(ne3370); ggSi1[hrde-1p::3xflag::gfp::hrde-1] II*	This study	MHE194
*C. elegans strain: gfp::prg-1; flag::mcherry::glh-1 I*	HC. Lee lab	HCL125
*C. elegans strain: csr-1(gcp027[mCherry::3xflag::1ha::csr-1]) IV; glh-1(sam24[glh-1::gfp::3xFlag]) I*	This study	MHE79
*C. elegans strain: piwi(gcp046[mCherry::prg-1]) I; mut-16(cmp3[mut-16::gfp::3xFLAG + loxP]) I*	This study	MHE147
*C. elegans strain: mut-16(cmp3[mut-16::gfp::3xFLAG + loxP]) I; csr-1(gcp027[mCherry::3xflag::1ha::csr-1] IV*	This study	MHE 93
*C. elegans strain: piwi(gcp038) I; mut-16(cmp3[mut-16::gfp::3xFLAG + loxP]) I; csr-1(gcp027[mCherry::3xflag::1ha::csr-1] IV*	This study	MHE117
*C. elegans strain: csr-1(gcp017[csr-1::3xFLAG::1HA])IV*	G. Cecere lab	MHE27
*C. elegans strain: csr-1(tm892)/nT1 [unc-?(n754) let-? qIs51] (IV;V)*	G. Cecere lab	MHE 20
*C. elegans strain: csr-1(gcp010)[D769A]/nT1 [unc-?(n754) let-? qIs51] (IV;V)*	G. Cecere lab	MHE 21
*C. elegans strain: glh-1(sam24[glh-1::gfp::3xFlag]) I*	D. Updike lab	DUP64
*C. elegans strain: glh-1(sam24[glh-1::gfp::3xFlag]) I; csr-1(tm892)/nT1 [unc-?(n754) let-? qIs51] (IV;V)*	This study	MHE 48
*C. elegans strain: glh-1(sam24[glh-1::gfp::3xFlag]) I; csr-1(gcp017[csr-1::3xFLAG::1HA];gcp022[D769A]/nT1[unc-?(n754) let-? qIs51] (IV;V)*	This study	MHE 49
*C. elegans strain: csr-1(gcp043[αN::3xFLAG::HA::CSR-1]) IV*	This study	MHE131
*C. elegans strain: ZK795.2(gcp064[zk795.2::3UTR_5boxb]) IV*	This study	MHE186
*C. elegans strain: ZK795.2(gcp066[zk795.2::3UTR_5boxb]) IV; csr-1(gcp043[αN::3xFLAG::HA::CSR-1]) IV*	This study	MHE188
*C. elegans strain: ZK795.2(gcp067[intronic_zk795.2::5boxb]) IV*	This study	MHE191
*C. elegans strain: ZK795.2(gcp067[intronic_zk795.2::5boxb]) IV; csr-1(gcp043[αN::3xFLAG::HA::CSR-1]) IV*	This study	MHE197
*C. elegans strain: hsp-90(gcp068[Phsp-90::αN::3xFLAG::mCherry] V*	This study	MHE201
*C. elegans strain: ZK795.2(gcp064[zk795.2::3UTR_5boxb]) IV; hsp-90(gcpp069[Phsp-90::αN::3xFLAG::mCherry]) V*	This study	MHE202

**Recombinant DNA**		

pJJR82 (EGFPˆSECˆ3xFlag)	Mike Boxem lab	Addgene plasmid # 75027; http://n2t.net/addgene:75027; RRID:Addgene_75027
pJJR83 (mCherryˆSECˆ3xFlag)	Mike Boxem lab	Addgene plasmid # 75028; http://n2t.net/addgene:75028; RRID:Addgene_75028
pFA6a-5BoxB-hphMX6 (5boxb)	Marc Bühler lab	(Bühler et al., 2006)
pRF4::rol-6(su1006)	(Mello et al., 1991)	N/A

**Software and algorithms**		

Integrative Genomics Viewer	http://www.broadinstitute.org/igv/	RRID:SCR_011793
Custom scripts and workflows for sequencing analysis	https://gitlab.pasteur.fr/bli/bioinfo_utils	DOI:10.5281/zenodo.5720645
Deeptools	https://deeptools.readthedocs.io/en/develop/	RRID:SCR_016366
Fiji	https://fiji.sc/	RRID:SCR_002285
StackReg (Fiji plugin)	(Thévenaz et al., 1998)	http://bigwww.epfl.ch/thevenaz/stackreg/
Oligo melting Python package	https://zenodo.org/record/4593033	DOI:10.5281/zenodo.4593033
iFISH probe design (ifpd)	https://ggirelli.github.io/iFISH-probe-design/	DOI:10.5281/zenodo.5584634
ImJoy	https://imjoy.io/#/	RRID:SCR_020935
Analysis workflows for smFISH image quantifications	https://github.com/fish-quant/fq-imjoy	DOI:10.5281/zenodo.5718531
Source code to analyze RNA localization in *C. elegans* germlines	https://github.com/muellerflorian/cornes-rna-loc	DOI:10.5281/zenodo.5718481

### Resource availability

#### Lead contact

Further information and requests for resources and reagents should be directed to and fulfilled by the lead contact, Germano Cecere (germano.cecere@pasteur.fr).

#### Materials availability

Strains generated in this study are available upon request from the authors.

### Experimental model and subject details

All strains used in this study are listed in the key resources table. Some strains were provided by the Caenorhabditis Genetics Center, which is funded by NIH Office of Research Infrastructure Programs (P40 OD010440). Strains were maintained at 20°C using standard methods (Stiernagle, 2006). Bristol N2 was used as the wild-type reference strain.

### Method details

#### Genome editing

##### Generation of CRISPR-Cas9 alleles

Cas9-guide RNA (gRNA) ribonucleoprotein complexes were microinjected into the hermaphrodite syncytial gonad (Paix et al., 2015). gRNA design and *in vitro* synthesis were done following the protocol detailed in (Barucci et al., 2020). For single-nucleotide modifications or small tag edits (e.g. *λN* tag), we used single-stranded DNA oligonucleotides ordered from IDT as standard 4 nM ultramer oligos. In the case of larger edits, such as fluorescent protein tag sequences, we generated double-stranded DNA repair templates by PCR amplifying eGFP or mCherry sequences from PJJR82 and PJJR83 plasmids (provided by the laboratory of M. Boxem). Silent mutations were included where necessary in the repair templates to prevent Cas9 cleavage. Mix concentrations were adapted from (Dokshin et al., 2018). In brief, 10 μl mixes typically contained the following final concentrations: 0,1μg/uL Cas9-NLS protein (TrueCut V2, Invitrogen), 100 ng/μl *in vitro* transcribed target-gene gRNA, 80ng/μl of target-gene ssODN repair template or 300ng/μl target-gene double-stranded DNA repair template and 80ng/uL pRF4::rol-6(su1006) plasmid (roller marker) (Mello et al., 1991). Cas9 and the target-gene gRNA were pre-incubated 10–15 min at 37°C before adding the other components to the mixture. dsDNA repair templates were subjected to a melting/annealing step (Dokshin et al., 2018) before addition to the final mix. *Screening and validation of CRISPR-Cas9 alleles. De novo* null *piwi* mutations were generated by introducing a premature STOP codon previously shown to completely abolish endogenous PIWI protein expression (Barucci et al., 2020). All strains were verified by sequencing of the edited locus. Sequences for gRNAs, single-stranded DNA, and double-stranded DNA repair templates and primers used for genotyping are available in Table S1.

#### Worm population sorting

To obtain large populations of precise developmentally staged worms for genome-wide approaches, we set up a sorting approach using a COPAS Biosorter (Union Biometrica). Eggs were collected by hypochlorite treatment, and synchronous populations of worms were grown for different time periods at 20 °C on OP-50 *E. coli* at a density of approximately 40,000 animals per Petri dish (15 cm). Depending on the developmental stage needed, synchronized worm populations were grown for ∼38 h (for early L4), 44 h (for late L4), and/or 48 h after hatching (for young adults). Synchronized populations were analyzed in the COPAS Biosorter based on optical density (optical extinction, EXT) and axial length (time of flight, TOF), and specific gates were designed for every developmental timepoint. The precision of the designed gates was evaluated by microscope visualization (DIC) of sorted samples. Morphological features of the vulva were used to define the accuracy of the designed sorting gates by counting the percentage of worms in a particular developmental stage. Note: Due to stochastic variability in growth timings across experiments, gating values had to be adjusted accordingly when needed to obtain a reproducible and homogeneous enrichment of specific developmental stages at the required timepoints. For mutants displaying strong developmental defects such as *rde-3*, growth timing and gating values were systematically adapted accordingly, and developmental stages scored based on morphological features of the vulva.

To apply this method using homozygous lethal *csr-1* mutants, we balanced *csr-1(KO)* and *csr-1(D769A)* strains using the nT1[qIs51] (IV;V) balancer, which contains a recessive lethal marker that causes embryonic lethality of homozygous balanced worms. In addition, it carries balancer-associated GFP transgenes that enable the visual identification of heterozygous animals (Edgley et al., 2006). Sorting of precisely staged homozygous animals was done by applying a non- GFP gate on previously developmentally gated populations. GFP gate precision was also determined by examining sorted populations, and an average sample purity of >94% (*csr-1* homozygous mutants) was obtained among all biological replicates used for sequencing.

#### RNA extraction

Synchronous or sorted worm populations were frozen in dry ice with TRI Reagent (Invitrogen) for total RNA extraction. After five repetitions of freeze and thaw, total RNA was isolated according to the manufacturer’s instructions. For RNA extraction after IP, TRI Reagent was directly added to beads. For RNA used for RNA-seq or RT-qPCR, DNase treatment was performed using a maximum of 10 μg RNA treated with 2 U Turbo DNase (Ambion) at 37 °C for 30 min followed by acid phenol extraction and ethanol precipitation. An Agilent 2200 TapeStation System was used to evaluate the RIN indexes of all RNA preps, and only samples with RNA integrity number (RIN)  > 8 were used for downstream applications.

#### IP- sRNA-seq

IP was performed using ∼10,000 synchronized sorted worms for 3xFLAG::CSR-1 or ∼70,000 for HRDE-1::GFP. A *de novo piwi* knockout mutation was generated by CRISPR/Cas9 on a strain carrying an HRDE-1::GFP reporter, and samples for IP were obtained from two independent lines 5 to 6 generations after *piwi* homozygosis. Worms were lysed in small RNA IP buffer (50 mM HEPES pH 7.5, 500 mM NaCl, 5 mM MgCl_2_, 1 % NP-40, 10 % glycerol, 1x Halt protease inhibitors and RNaseIn 40 U/mL), using a chilled metal dounce. Crude lysates were cleared of debris by centrifuging at 18,000 g at 4°C for 10 min. 10 % of the extract was saved as input, and total RNA was extracted using TRI reagent (Invitrogen). The rest of the extract was incubated with 15 μl of packed Anti-FLAG M2 Magnetic Agarose Beads (Sigma M8823) or 25 μl GFP-Trap Agarose Beads (Chromotek) for FLAG-CSR-1 or GFP-HRDE-1 respectively, for 1 h at 4°C. After four washes of the beads with the small RNA IP buffer, the RNA bound to bait was extracted by adding TRI reagent to beads as described above. The library preparation was performed essentially as described previously (Barucci et al., 2020). Amplified libraries were multiplexed to purify further using PippinPrep DNA size selection with 3% gel cassettes and the following parameters for the selection: BP start (115); BP end (165). The purified libraries were quantified using the Qubit Fluorometer High Sensitivity dsDNA assay kit (Thermo Fisher Scientific, Q32851) and sequenced on a NextSeq-500 Illumina platform using the NextSeq 500/550 High Output v2 kit 75 cycles (FC-404-2005).

#### GRO-seq

One thousand sorted worms were collected, and the Nuclear Run-on reaction was performed by incorporating 1 mM Bio-11-UTP, followed by RNA extraction and biotinylated nascent RNA enrichment, as described previously (Quarato et al., 2021). Libraries were prepared by repairing 5′-OH of fragmented RNAs with T4 Polynucleotide Kinase (New England Biolabs), followed by 3′ and 5′ adaptor ligation as described in (Quarato et al., 2021). Adaptor ligated RNA was reverse transcribed using SuperScript IV Reverse Transcriptase (Thermo Fisher Scientific) following manufacturer conditions except that reaction was incubated for 1h at 50°C and 10 min at 80 °C. cDNA was PCR amplified with specific primers using NEBNext® Ultra™ II Q5® Master Mix 2x (New England Biolabs) for 18–20 cycles. Libraries were analyzed on Agilent 2200 TapeStation System using high sensitivity D1000 screentapes and quantified using the Qubit Fluorometer High Sensitivity dsDNA assay kit (Thermo Fisher Scientific, Q32851). Multiplexed libraries were sequenced on a NextSeq-500 Illumina platform using the NextSeq 500/550 High Output v2 kit 75 cycles (FC-404–2005).

#### Strand-specific RNA-seq library preparation

DNase-treated total RNA with RIN > 8 was used to prepare strand-specific RNA libraries. Ribosomal and mitochondrial rRNAs were depleted using a custom RNAse-H-based method to degrade rRNAs using complementary oligos, as described in (Barucci et al., 2020).

Strand-specific RNA libraries were prepared using at least 100 ng of rRNA depleted RNAs using NEBNext Ultra II Directional RNA Library Prep Kit for Illumina (E7760S). RNA libraries were analyzed on Agilent 2200 TapeStation System using high sensitivity D1000 screentapes and quantified using the Qubit Fluorometer High Sensitivity dsDNA assay kit (Thermo Fisher Scientific, Q32851). Multiplexed libraries were sequenced on a NextSeq-500 Illumina platform using the NextSeq 500/550 High Output v2 kit 75 cycles (FC-404–2005).

#### Sequencing data analyses

Analysis for RNA-seq, sRNA-seq, and GRO-seq have been performed as previously described (Barucci et al., 2020; Quarato et al., 2021). Unless otherwise stated, computations were done using Python and UNIX utilities, either as standalone scripts or as steps implemented in a Snakemake workflow (Köster and Rahmann, 2012; Mölder et al., 2021). The scripts and workflows are available at https://gitlab.pasteur.fr/bli/bioinfo_utils.

#### Profile of 22G-RNAs across piRNA target sites

Annotated sequences of piRNAs (21U-RNAs) were used to predict their targets as described by (Zhang et al., 2018) using the following stringent criteria for piRNA targeting: up to one GU wobble pair was allowed in the seed region, and overall up to two mismatches and an additional GU mismatch were allowed. In addition, the mismatch at the first nucleotide of a piRNA is not counted/considered. The density of antisense 22G-RNAs within a 200 nt window around predicted 21U-RNA target sites was determined using RPM from sRNA-seq analysis by summarizing normalized coverage information (taken from bigwig files and averaged across replicates) along with spermatogenic HRDE-1 targets and non-targets using deeptools (Ramírez et al., 2016).

#### Tissue enrichment and calculation of enrichment factor

Tissue enrichment of spermatogenic HRDE-1 targets was calculated using WormBase Enrichment Suite (Angeles-Albores et al., 2018). Spermatogenic HRDE-1 targets in gene expression categories of sperm-specific and germline-specific (Ebbing et al., 2018) have been calculated as follows, considering the total number of *C. elegans* protein-coding genes (20,447): [(Spermatogenic HRDE-1 targets) × (number of genes in the gene expression category)/total number of *C. elegans* protein-coding genes]. Enrichment has been calculated as the ratio between observed and predicted genes for each gene expression category.

#### polyUG RNA detection

Gene-specific polyUG RNAs were detected by polyUG PCR as described in (Shukla et al., 2020). Essentially, 5μg of total RNA was used in a reverse transcription reaction using 1pmol of polyUG-specific RT and M-MLV reverse transcriptase (Invitrogen). 1uL of cDNA was used in a first PCR (20μL - 25 cycles) using DreamTaq DNA polymerase (ThermoFisher) and primers listed in Table S2. Next, PCRs were diluted at 1:100 and 1μL used for a second PCR (50μL volume – 30 cycles) using primers listed in Table S2. The original raw data for the blots shown in Figure 2 can be found at https://data.mendeley.com/datasets/tnmxzg8wjj/1.

#### Confocal live imaging

Larvae and adult worms were immobilized in M9 with 10-20mM levamisole and mounted on 2% agarose pad glass slides. Germlines were imaged using a ZEISS LSM 700 microscope or a ZEISS LSM 880 AxioObserver with a Plan-Apochromat 63x/1.40 Oil M27 objective. Images were obtained using the ZEISS ZEN microscope software and processed using ImageJ v.2.0.0.

#### Quantification of MUT-16 foci density

Germ cell nuclei from ten different germlines were live imaged using a Plan-Apochromat 100x/1.46 Oil DIC objective. Multiple z-stack images were aligned using the FIJI plugin StackReg (Thévenaz et al., 1998). Foci number was counted on maximum intensity Z projections after applying a thresholding step from FIJI in manually defined regions of the germline corresponding to distal or pachytene. The number of foci was normalized to the area of the defined region to obtain a density value.

#### Quantification of distances between condensates

Germ cell nuclei from ten different germlines were live imaged using a Plan-Apochromat 100x/1.46 Oil DIC objective. Confocal z-stack images were aligned using the FIJI plugin StackReg (Thévenaz et al., 1998) and processed with the 3D analysis software Imaris. Condensates were 3D segmented in each channel and centers automatically detected using the spot function of IMARIS. We manually measured, around each nucleus, the distance between adjacent spots of different channels using the IMARIS ruler. At least ten pairs of condensates were selected from different regions of each individual germline. As an experimental measure of chromatic shift between different fluorescent channels, 0.1 μm Tetraspeck beads were imaged, and the 3D distance between centers was calculated. Additionally, an unbiased co-localization analysis was performed automatically with a custom-written Python script. In brief, the Hungarian algorithm (SciPy function optimize.linear_sum_assignment) was used to identify adjacent spots in both channels. This algorithm finds the globally optimal solution for the entire image and provides for each pair their 3D distance. The percentage of matched pairs is then reported as a function of different distance thresholds tested.

#### Single-molecule fluorescent in-situ hybridization (smFISH)

Whole worm smFISH was essentially performed as described in(Ji and van Oudenaarden, 2012) using unlabeled primary probes and fluorescently labeled secondary detector oligonucleotides (Tsanov et al., 2016). The intronic probes were designed by extracting all the available 30-mers from the target intronic sequences, retaining only those with GC-content between 35 and 85%. The pool of oligonucleotides obtained from this step was further filtered by discarding sequences containing homopolymers (>=7), having at least one off-target (<= 5 mismatches) across the *C. elegans* genome (WBcel235 assembly) and deviation from the pool’s average melting temperature >10 °C. The melting temperature was calculated using the *oligo-melting* python package (https://zenodo.org/record/4593033) with parameters ‘*-t DNA:RNA -o 0.05e-6 -n 1.04 -f 25’*. The final selection of the oligonucleotides that constitute the probe targeting each intronic region was performed using iFISH probe design (*ifpd*) (https://ggirelli.github.io/iFISH-probe-design/) using default parameters. Multiple stack images were acquired for different channels on a Zeiss Axio Imager M2 microscope equipped with a Princeton Instrument PIXIS 1024 camera, using Plan-Apochromat 100x or 63x/1.40 Oil DIC objectives. Images were processed using an in-house deconvolution software before the quantification of the smFISH signal.

#### FISH signal quantification along the germline

Detection of RNAs in 3D images was performed with a standard spot detection approach implemented in Python, which is interfaced with the web application ImJoy (Imbert et al., 2021). Briefly, images were filtered with a 3D Laplacian of Gaussian filter (LoG), and RNAs were detected with a local maximum detection. The analysis software was also used to manually draw a central axis through the germline. Each RNA is assigned to the closest point on the axes in a defined threshold region (see schematic in Figure 4D). The distances along the axes are calculated and centered around the starting position (0) that corresponds to the germline loop (defined as the most left point in the axis). Positive distances correspond to the proximal part and negative distances to the most distal part. A post-processing script calculates RNA enrichment along this axis. RNA counts were binned, and for each experimental condition, the mean +/- standard deviation of 5 to 10 germlines is reported. Source code with detailed manuals and test data is available on GitHub. Code for smFISH analysis is available at https://github.com/fish-quant/fq-imjoy, code for post-processing scripts is available at https://github.com/muellerflorian/cornes-rna-loc.

#### Sperm activation assay

L4 males were isolated and kept overnight at 20°C for 24hours. Spermatids were isolated from at least 10 males were washed and dissected in sperm medium (50mM HEPES pH7.8, 50mM NaCl, 25mM KCl, 5mM CaCl2, and 1mM MgSO4) completed with 20μg/mL pronase E, on 3-well 14mm diagnostic slides. Spermatids were imaged 30 min after dissection and were scored into three categories: spermatids with normal pseudopods (wild-type), arrested spermatids with irregular pseudopods (irregular), and arrested spermatids with no pseudopods (inactivated).

#### Endogenous CSR-1 tethering assay

Bacteriophage Lambda anti-termination peptide (λN) and *5 boxb* hairpin sequences were integrated by CRISPR-Cas9 at selected genetic loci.

##### Generation of *λN*::3xflag::ha::csr-1 strain

a *C. elegans* codon-optimized version of the λN anti-termination peptide sequence used in (Bühler et al., 2006), which also contains a Pro-Pro-Leu linker, was included in a single-stranded DNA oligonucleotide to be used as a repair template. A specific gRNA was used to insert *λN* tag immediately upstream the first flag sequence of a previously generated *3xflag::ha::csr-1 strain*, tagging the two isoforms of CSR-1 (Barucci et al., 2020). Similarly, a *λN::3xflag* tag was introduced after the start codon of a mCherry sequence in an endogenous transcriptional reporter strain *Phsp-90::mCherry*::SL2. *Generation of ZK795.2::5boxB strains*: 5boxb hairpin sequences were PCR amplified from the plasmid pFA6a-5BoxB-hphMX6 (kindly provided by the M. Bühler lab) with primers containing 33bp homology arms to generate a dsDNA repair template to be integrated either in the second intron or after the stop codon of the ZK795.2 gene in wild-type and *λN::3xflag::ha::csr-1* strains. To evaluate the tethering of mCHERRY to ZK795.2 mRNA, we crossed the strains carrying the λ*N::3xflag::mCherry* and *ZK795.2::5boxb* alleles.

#### RNA-IP

The synchronous population of 80,000 *λN::3xflag::ha::csr-1* or *λN::3xflag::mCherry* worms were collected 44 h after hatching and suspended in extraction buffer (50 mM HEPES pH 7.5, 300 mM NaCl, 5 mM MgCl_2_, 10% glycerol, 0.25% NP-40, Halt protease inhibitor cocktails (Thermo Fisher Scientific), Samples were treated by at least 50 strokes using a metal dounce on ice and crude protein extracts were centrifuged at 12,000 r.p.m. at 4 °C for 10 min. Protein concentration was quantified using the Bradford assay, and 1 mg of protein extract was incubated with 15 μl of packed anti-Flag M2 magnetic agarose beads (Sigma, M8823) for 2  at 4 °C. After four washes with extraction buffer, 1mL of TRI reagent (Invitrogen) was directly added to the magnetic beads to extract the immunoprecipitated RNAs. 1mL of TRI reagent was also added to 10% of protein extract before the IP (input). The resulting isolated RNA was analyzed using RT–qPCR to quantify mRNA levels.

#### RT-qPCR

Reverse transcription was performed using M-MLV reverse transcriptase (Invitrogen), and qPCR was performed using Applied Biosystems Power up SYBR Green PCR Master mix and using an Applied Biosystems QuantStudio 3 Real-Time PCR System. Primers used for qPCR are listed in Table S3.

#### Gene lists

The gene lists used are provided in Table S4.

### Quantification and statistical analysis

Measurements were sampled from individual biological replicates. RNA-seq experiments, GRO-seq and IP-sRNA-seq were performed independently using at least two biological replicates consisting in sorted synchronized worm populations (>1000 worms). RT-qPCRs for gene expression changes were performed in three independent biological replicates unless otherwise stated. When groups of continuous data are compared, two-tailed t tests with unequal variance were performed; exact p values are shown in every graph. Box plots display median, first, and third quartiles, and 90^th^/10^th^ percentile values. Otherwise, statistical tests and sample sizes used are detailed in the corresponding Figure legends; graphs and calculations were performed using GraphPad Prism 8. No statistical methods were used to predetermine sample sizes.

## Data Availability

• All the sequencing data are available at the Gene Expression Omnibus (GEO) under accession code GSE157319. Original polyUG PCR gel images have been deposited at Mendeley and are publicly available at the DOI listed in the key resources table. • Custom code and scripts are available from key resources and methods details. DOIs are listed in the key resources table. • All other data supporting the findings of this study are available from the corresponding author upon request.

## Supplemental information

Document S1. Figures S1–S7

Table S1. Sequences for gRNAs, single-stranded DNA, double-stranded DNA repair templates and primers used for genotyping, related to Figures 1, 2, 7, and S2

Table S2. Primers used for polyUG PCRs, related to Figure 2

Table S3. Primers used for qPCR, related to Figures 2 and 7

Table S4. Gene lists generated and used in this study, related to Figure 1

Document S2. Article plus supplemental information
