# Supplementary material for: piRNAs initiate transcriptional silencing of spermatogenic genes during C. elegans germline development
Source: Dev Cell. 2022 Jan 24;57(2):180–196.e7. doi: 10.1016/j.devcel.2021.11.025 (PMC8796119; doi:10.1016/j.devcel.2021.11.025)
Supplement: Document S1. Figures S1–S7 [file mmc1.pdf]

Developmental Cell, Volume *57*

Supplemental information

piRNAs initiate transcriptional silencing of spermatogenic genes during *C. elegans* germline development

Eric Cornes, Loan Bourdon, Meetali Singh, Florian Mueller, Piergiuseppe Quarato, Erik Wernersson, Magda Bienko, Blaise Li, and Germano Cecere

SUPPLEMENTAL FIGURES

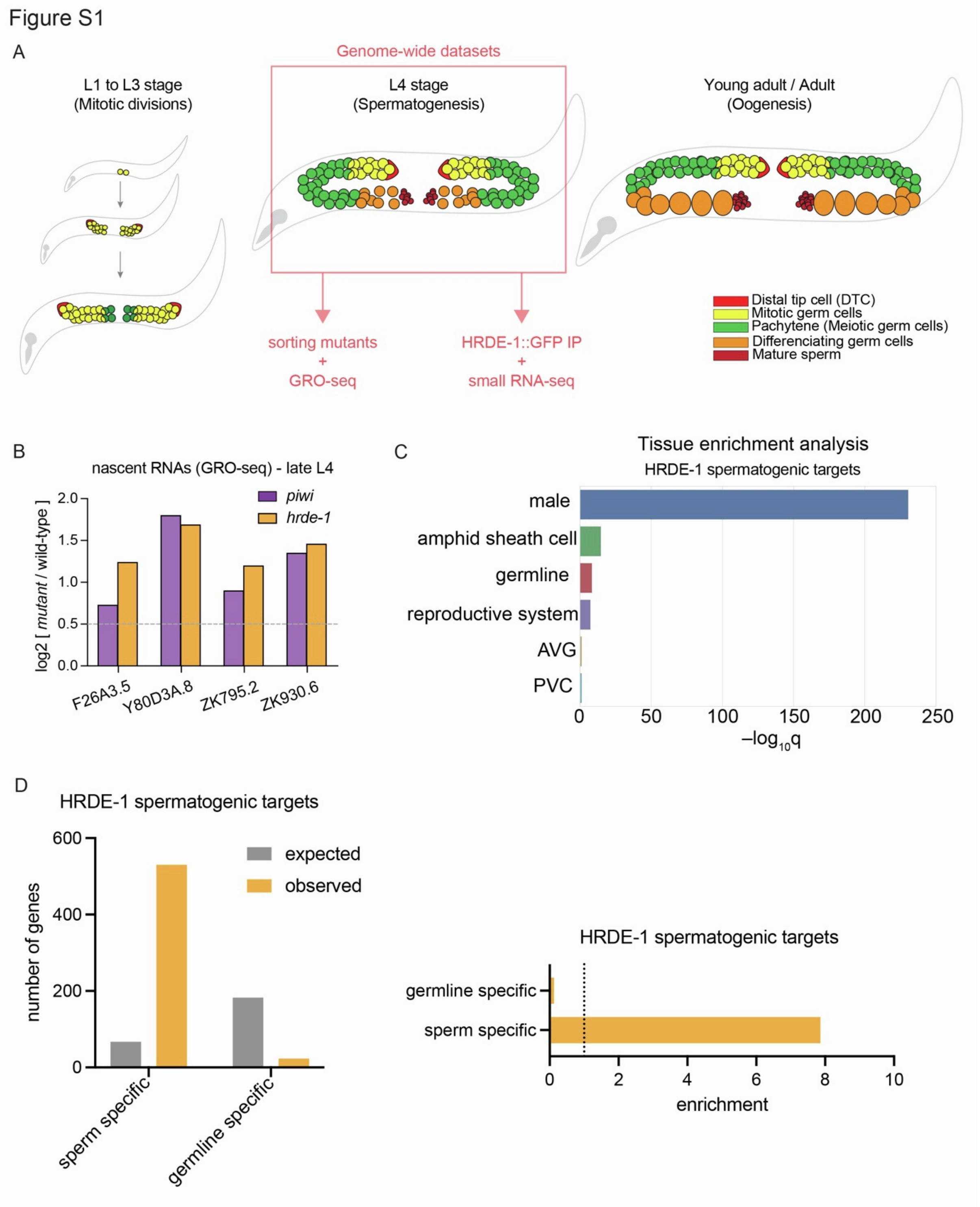

Figure S1. Upregulation of piRNA targets in the *C. elegans* hermaphrodite germline during spermatogenesis. Related to Figure 1.

(A) Schematic representation of the *C. elegans* germline development. Hermaphrodite gonads develop from two primordial germ cells as two symmetric gonadal arms. Along the three first

larval stages (L1 – L3), germ cells derived from a stem cell niche associated with the distal tip cell (DTC) (red) progress mitotically towards the proximal end of the syncytial gonad (yellow). During the L3 stage, germ cells enter meiosis proximally and reach the pachytene stage (green) at the end of L3. During L4, the first cells that enter meiosis undergo spermatogenesis (orange), whereas germ cells entering meiosis during the later L4 stage will become oocytes at the young adult stage (orange), only after sperm differentiation (dark red) has been completed. (B) $\log_2$ fold change in nascent RNAs (GRO-seq) for the spermatogenic piRNA targets highlighted in Figure 1D measured in late L4 sorted populations of *piwi* and *hrde-1* mutants compared to wild-type. (C) Tissue enrichment analysis for spermatogenic HRDE-1 targets. (D) Predicted and observed distribution (left) and enrichment (right) of spermatogenic HRDE-1 targets in different gene expression categories.

Figure S2

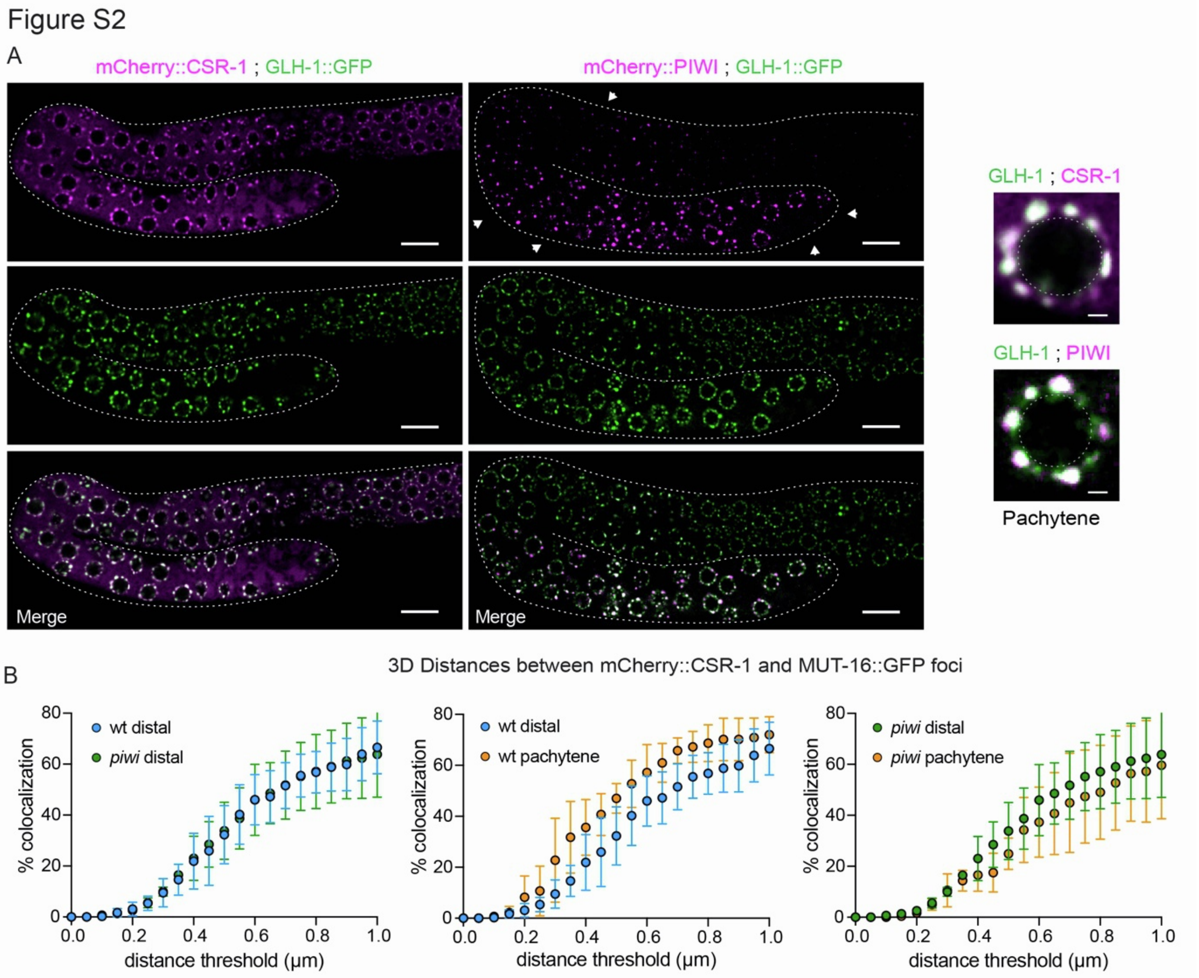

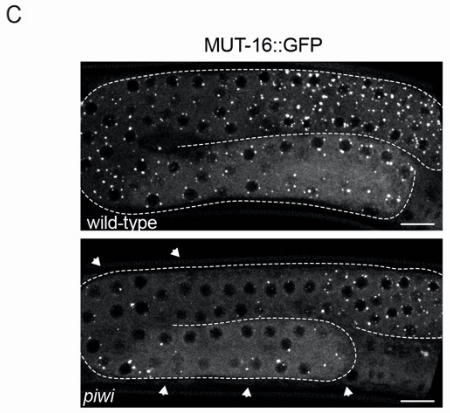

D

Analysis of PIWI interactors from PIWI IP mass spectometry
(Barucci et al., 2020)

PROTEIN	log_2 fold change / wild-type IP	p value	Protein Domain	Cellular localization
**PGL-1**	1.4	$3.7e^{-25}$	RGG-box Worm-specific protein	P granule
**PGL-2**	9.9	*	Worm-specific protein	P granule
**PGL-3**	2.3	$8.2e^{-24}$	RGG-box Worm-specific protein	P granule
**GLH-1**	3.2	$1.1e^{-36}$	DEAD-box RNA helicase *Vasa* homolog	P granule
**GLH-2**	1.2	0.008	DEAD-box RNA helicase *Vasa* homolog	P granule
**GLH-3**	9.9	*	DEAD-box RNA helicase *Vasa* homolog	P granule
**GLH-4**	4.1	$1.3e^{-25}$	DEAD-box RNA helicase *Vasa* homolog	P granule
**MUT-16**	9.9	*	Q/N Rich	Mutator foci
**RDE-3**	9.9	*	ribo-nucleotidyltransferase	Mutator foci
**CSR-1**	1.4	$7.6e^{-51}$	Argonaute	P granule
**HRDE-1**	1.9	$3.8e^{-14}$	Argonaute	Nucleus

Figure S2. Germline localization of AGO proteins and 22G-RNA biogenesis machinery during spermatogenesis. Related to Figure 2.

(A) (Left) Panels show a single confocal plane of live germlines expressing fluorescently tagged endogenous CSR-1 and PIWI proteins during the L4 stage. GLH-1 was used to visualize

P granules along with the germline tissue. Scale bars 10μm. (Right) Fluorescent micrographs of a single pachytene germ-cell nucleus from animals expressing the indicated fluorescent proteins. Scale bars 1μm. (B) Unbiased measurement of the 3D distances between MUT-16::GFP and mCherry::CSR-1 condensates (see Star Methods). Plots show the percentage of mCherry::CSR-1 and MUT-16::GFP co-localization events as a function of different distance thresholds tested. The co-localization curves in the distal region of the germline from wild-type and *piwi* mutants show a very similar distribution. However, we observe a faster increase in pachytene co-localization curve than distal regions, specifically in wild-type germlines, similar to the results obtained from manually measured distance among foci and reflecting the inclusion of MUT-16 into CSR-1 granules. Mean and standard deviation of measurements from 3 different germlines per genotype. (Number of MUT-16 foci analyzed per sample; wild-type pachytene: 101>n>42, wild-type distal: 190>n>105, *piwi* mutant pachytene: 75>n>25, *piwi* mutant distal: 109>n>60.) (C) Panels show a confocal z-stack of germline surfaces from live animals expressing MUT-16::GFP during the L4 stage. Scale bars 10μm. (D) Proteomic analysis of the PIWI complex. Only a selection of PIWI-interacting proteins corresponding to known germ-granule components, RNAi factors, and argonaute proteins is shown. Asterisks mark interactors for which no p-value could be calculated due to the absence of peptides detected in control IPs.

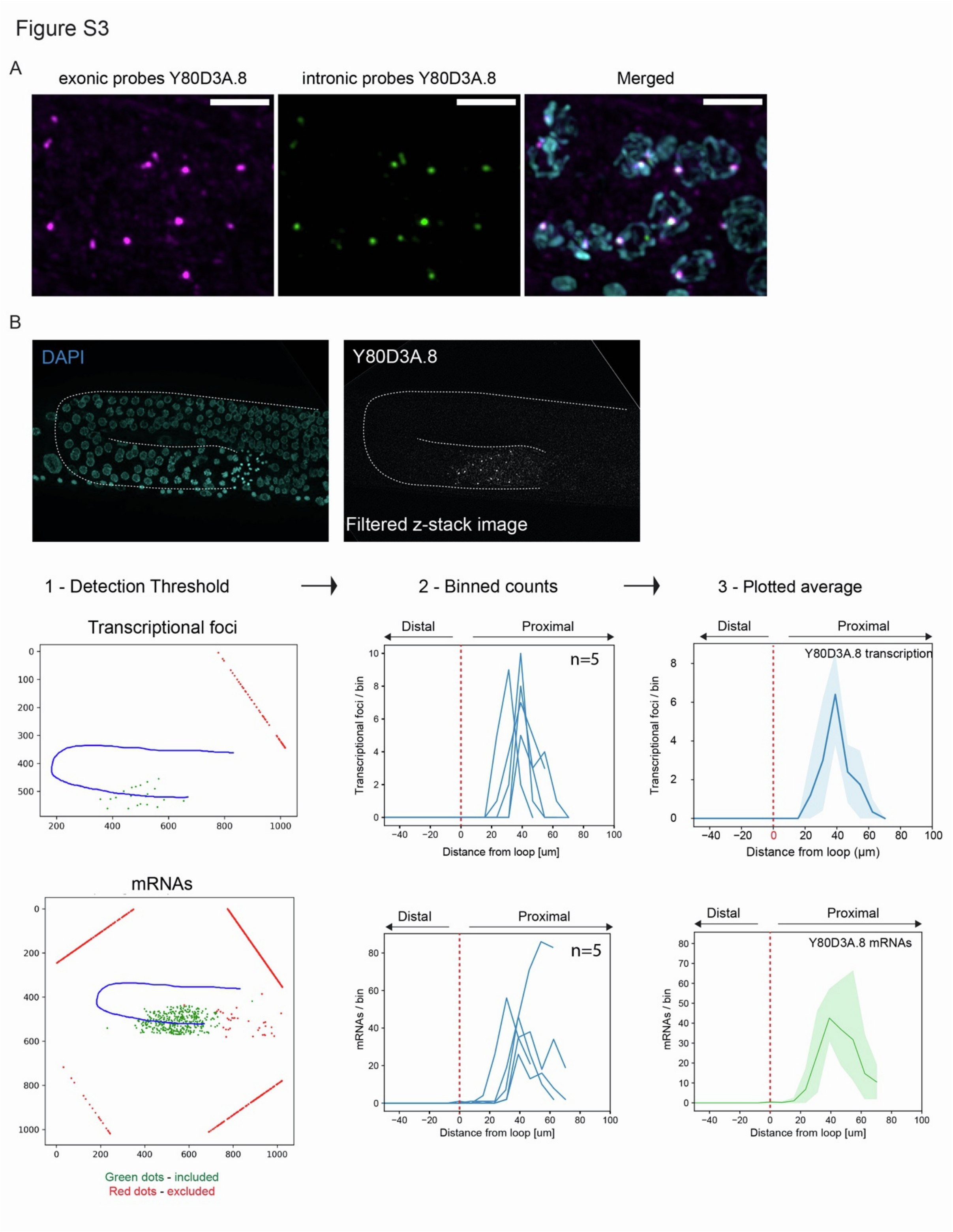

Figure S3. Quantification of Y80D3A.8 transcriptional and mRNA smFISH signal. Related to Figure 3.

(A) Y80D3A.8 smFISH in a wild-type germline, detail of the proximal pachytene region. Exonic probes (magenta), intronic probes (green), nuclei are visualized with DAPI (cyan).

Signal from exonic probes show dim cytoplasmic mRNA signal and nuclear bright spots. Signal from intronic probes show nuclear bright spots overlapping with the exonic probe signal (white) in the nucleus. Scale bars 5μm. (B) Example of the pipeline workflow used for quantification of Y80D3A.8 smFISH signal. Filtered 3d stacks were used to detect either individual mRNAs or active transcriptional sites by adjusting the intensity thresholds for spot detection. The analysis software was also used to manually draw a central axis through the germline (blue line) using the DAPI channel as a reference to detect the most proximal differentiating spermatocyte. The remove false-positive detections in the background, only spots within 80 pixels of the manually drawn axis were used for further analysis. A postprocessing script calculates RNA enrichment along this axis, by assigning each RNA to the closest pixel on the axis. RNA counts are binned for better representation, and for each experimental condition, the mean +/- standard deviation of at least 5 germlines is reported.

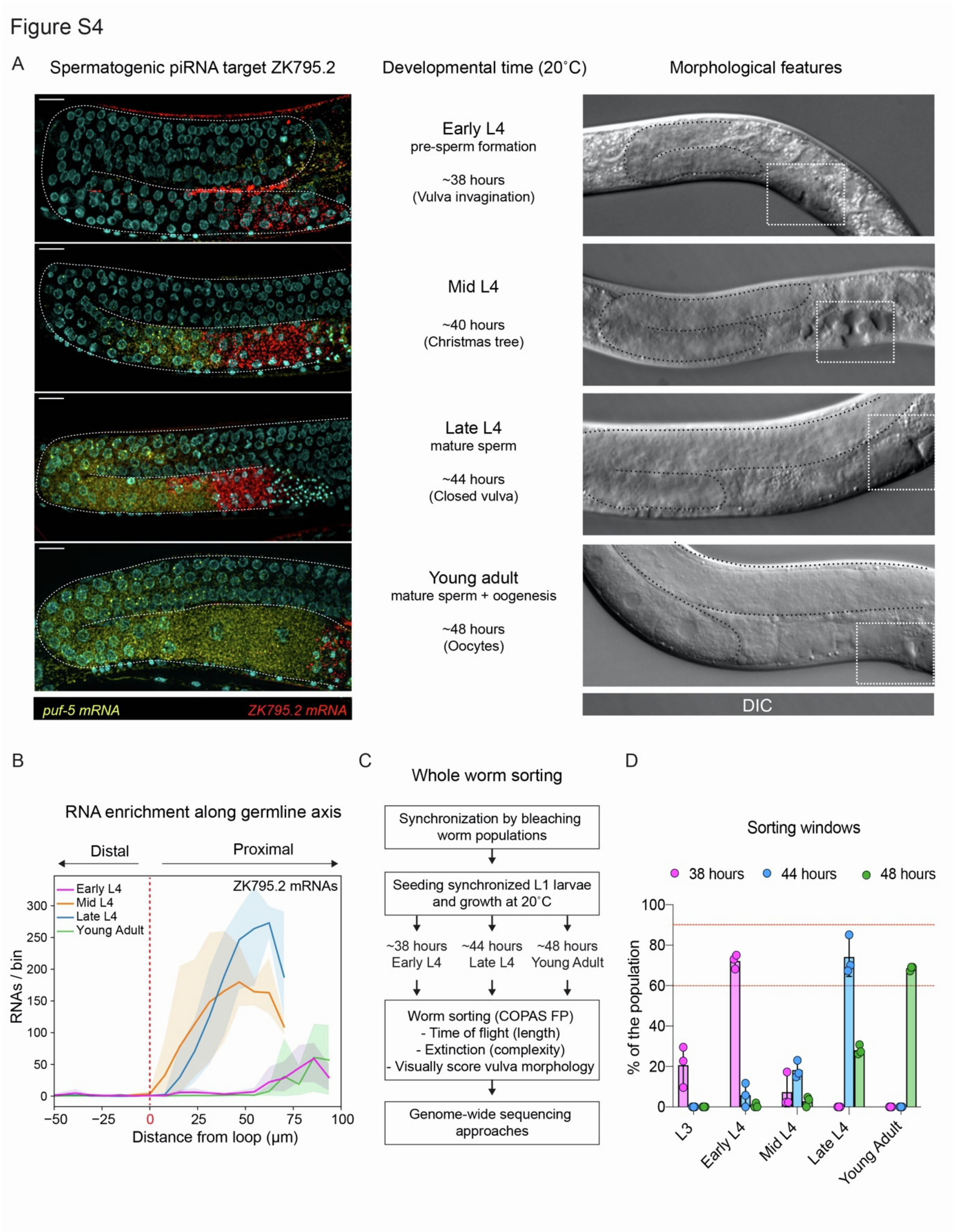

Figure S4. A worm sorting strategy to study spermatogenic gene expression dynamics during development. Related to Figure 4.

(A) Spermatogenic and oogenic gene expression can be tracked by smFISH using probes against sperm- (ZK795.2) or oogenic-enriched (*puf-5*) mRNAs. Panels show z-stacks from

individual wild-type germlines at the indicated developmental timepoints. ZK795.2 mRNAs (red), *puf-5* mRNAs (yellow), and DNA visualized with DAPI (cyan). At the early L4 stage (~38 hours post L1), an invagination that will give rise to the future vulva is observable in the center of the worm body cavity (white square), and ZK795.2 transcripts start to be detected at the most proximal part of the germline as a result of spermatogenic gene transcription in the pachytene region. During mid phases of L4 (~40 hours post L1), the vulval structure organizes in a particular shape, also known as the *Christmas tree*, the accumulation of ZK795.2 transcripts in the proximal region of the germline accompanies germ cell progression through meiosis towards sperm differentiation. In late L4 (~44 hours post-L1), the vulva structure is closed, and spermatogenesis is almost completed, with the presence of mature spermatids and residual bodies resulting from meiotic divisions (white asterisks). Once all sperm cells have been formed, residual ZK795.2 smFISH signal is detected, and oocyte differentiation begins. The presence of developing unfertilized oocytes (white asterisks) defines the young adult stage (~48 hours post L1). (B) Number of spermatogenic piRNA target (ZK795.2) mRNAs as a function of germline position at the indicated developmental timepoints. Average and standard deviation values measured from n = 5 germlines are shown. The red dashed line indicates the position of the germline loop. (C) Summary of whole worm sorting strategy to obtain large worm populations enriched in specific sub-stages associated with different steps of spermatogenesis (see Methods). (D) Percentages of developmental stages obtained from sorted populations at the indicated time points are shown. The bars indicate the median value, and error bars indicate the 95% confidence interval from 3 biologically independent experiments (n = 50 worms scored per biological replicate). Red lines delimitate the homogeneous range of enrichment in a particular developmental stage for each timepoint.

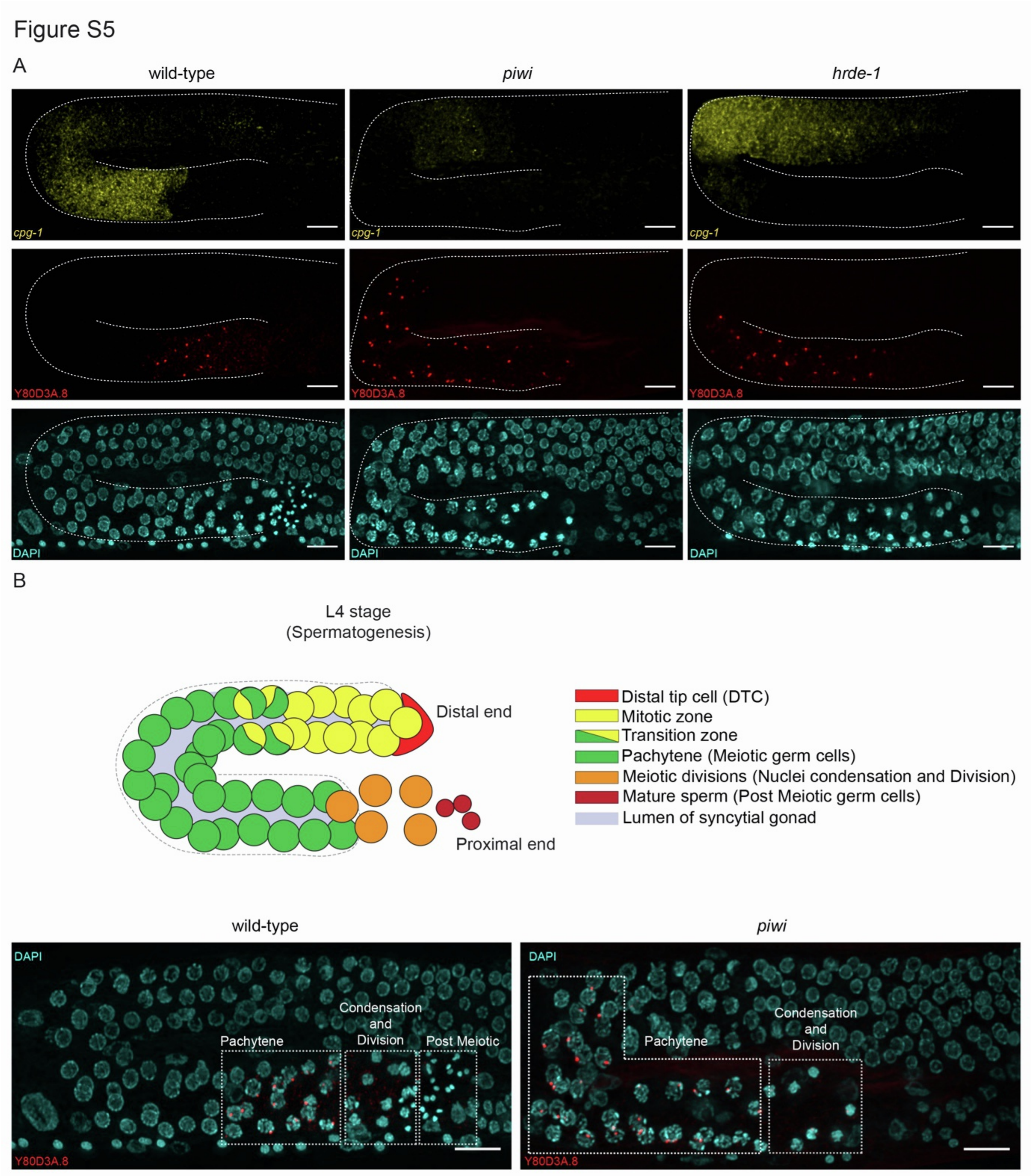

Figure S5. Increased number of pachytene cells transcribing spermatogenic genes in piRNA pathway mutants. Related to Figure 5.

(A) Visualization of a spermatogenic piRNA target (Y80D3A.8) mRNA expression by smFISH. Panels show z-stacks of late L4 germlines at the indicated genetic backgrounds. Y80D3A.8 mRNAs (red), *cpg-1* mRNAs (yellow), and DNA visualized with DAPI (cyan). Scale bars 10µm. (B) Schematic representation of a C. elegans hermaphrodite germline

undergoing spermatogenesis during the L4 stage. Germ cell nuclei (colored circles) migrate linearly from the distal to the proximal end of the germline syncytial tissue and progress through different cell-cycle phases before differentiating into mature sperm cells. Mitotic proliferation occurs in the most distal region of the germline and is influenced by the somatic distal tip cells (DTC). The earliest steps of the meiotic prophase start as germ cells migrate away from the distal tip cell and transit the transition zone. The transient transcription and translation of spermatogenic genes occur in the pachytene region. Spermatogenic differentiation requires global transcriptional repression and nuclear condensation, which occurs before detaching the differentiating germ cells from the syncytium to achieve the meiotic divisions and spermatid activation. (Bottom panels) z-stacks of late L4 germlines from wild-type and *piwi* mutants transcribing a spermatogenic piRNA target. Y80D3A.8 mRNAs (red) and DNA visualized with DAPI (cyan). Scale bars 10µm. Dashed white squares highlight different meiotic regions.

Figure S6

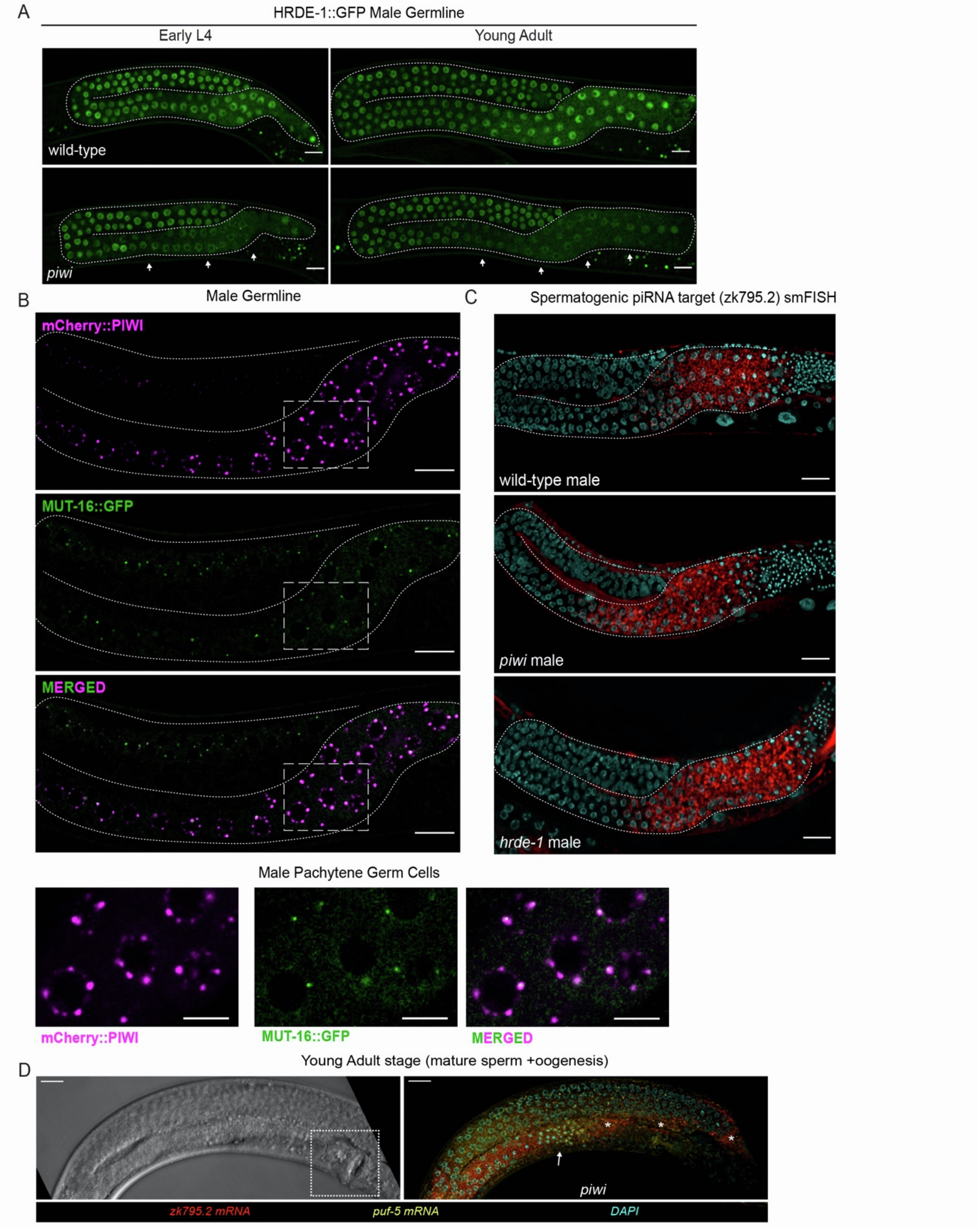

Figure S6. Repression of spermatogenic gene expression by piRNAs during spermatogenesis in the male germline. Related to Figure 6.

(A) Panels show a single confocal plane of live male germlines expressing an HRDE-1::GFP reporter at the indicated developmental timepoints and genetic backgrounds. Arrows indicate

pachytene-specific loss of nuclear HRDE-1 enrichment. Scale bars 10μm. (B) (Top panels) Panels showing a single confocal plane of live wild-type male germlines expressing mCherry::PIWI and MUT-16::GFP in wild-type during the L4 stage. Scale bars 10μm. (Bottom panels) Fluorescent micrograph of pachytene germ-cell nuclei from animals expressing the indicated fluorescent proteins. Scale bars 5μm. (C) smFISH of ZK795.2 in male germlines. Panels show z-stacks from male germlines from the indicated genetic backgrounds at the same stage of spermatogenesis. ZK795.2 mRNAs (red) and DNA visualized with DAPI (blue). Scale bars 10μm. (D) Enlarged spermatogenic ZK795.2 expression domain in Young Adult *piwi* mutant germlines. Left panel: DIC image. White dashed square highlights the presence of a fully formed vulva. Right panel: smFISH of the spermatogenic piRNA target ZK795.2 (red) and the oogenic enriched gene *puf-5* (yellow). DNA staining with DAPI (cyan). Arrows indicate mature sperm. Asterisks highlight regions with high background autofluorescence. Scale bars 15μm.

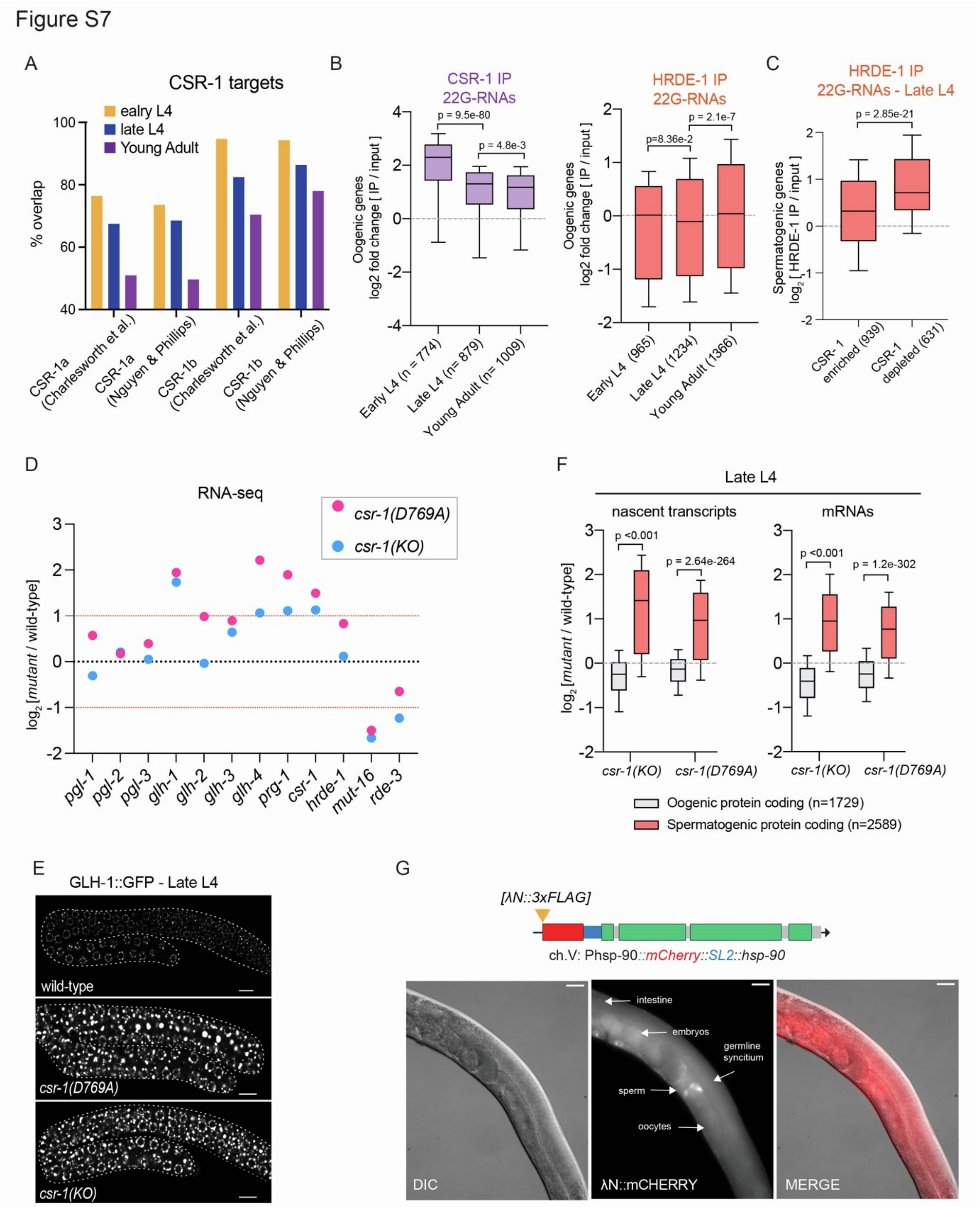

Figure S7. Targeting of spermatogenic genes by the germline AGO CSR-1. Related to Figure 7.

(A) Percentage of overlap between CSR-1 IP targets obtained in this study across sorted early L4, Late L4, and Young Adult stages with CSR-1a and CSR-1b isoform IPs from (Charlesworth et al., 2021; Nguyen and Phillips, 2021). (B) Box plots showing the $\log_2$ fold

change of 22G-RNAs (sRNA-seq) in HRDE-1 and CSR-1 IPs compared to input in wild-type animal populations at three developmental time points for oogenic enriched genes. Box plots display median (line), first and third quartiles (box), and $90^{th}/10^{th}$ percentile values (whiskers). Two-tailed P values were calculated using Mann–Whitney–Wilcoxon tests. (C) Box plots as in (B) showing the $\log_2$ fold change of 22G-RNAs (sRNA-seq) in HRDE-1 IPs compared to input in Late L4 wild-type animal populations for CSR-1 enriched or depleted spermatogenic genes. (D) $\log_2$ fold change in mRNAs (RNA-seq) of genes encoding for canonical P granule *(pgl-1, pgl-2, pgl-3, glh-1, glh-2, glh-3, glh-4)*, Argonaute *(prg-1, csr-1, hrde-1),* piRNA pathway *(mut-16),* and polyUG pathway (*rde-3*) proteins, measured in late L4 sorted populations of *csr-1* mutants compared to wild-type animals. (E) Live confocal images of the P granule reporter GLH-1::GFP in *csr-1(D769A)* and *csr-1(KO)* mutant germlines compared to wild-type. Scale bars, 10µm. (F) Box plots as in (B) showing the $\log_2$ fold change in spermatogenic (red) or oogenic enriched (gray) nascent RNAs (GRO-seq) or mRNAs (RNA-seq) in *csr-1(KO)* and *csr-1(D769A)* mutant worms versus wild-type at the late L4 stage. (G) Schematic representation of the λN-mCHERRY control strain used in the AGO tethering assay. An endogenous *Phsp-90::cherry* transcriptional reporter was made by CRISPR/Cas9 knock-in of a mCherry sequence followed by a *gpd-2* SL2 trans-splicing sequence. By this method, mCHERRY can be co-expressed independently to the endogenous HSP-90 protein. Using again CRISPR/Cas9 genome editing, we introduced a λN::3xflag tag after the START codon of a mCherry sequence. As a result, a functional λN::CHERRY protein was ubiquitously and highly expressed in somatic and germline tissues throughout worm development. Scale bars 25µm.
